# Vaccine-induced mouse antibodies targeting *Plasmodium falciparum* PfVFT antigen inhibit blood stages through multiple mechanisms

**DOI:** 10.1038/s41541-026-01433-9

**Published:** 2026-03-30

**Authors:** Yun Shan Goh, Haitong Mao, Pei Xiang Hor, Chiew Yee Loh, Zi Wei Chang, Cornelus C. Hermsen, Francois Nosten, Robert W. Sauerwein, Laurent Rénia

**Affiliations:** 1 A*STAR Infectious Diseases Labs (A*STAR ID Labs), Agency for Science, Technology and Research (A*STAR), Singapore, Singapore; 2 Department of Medical Microbiology, Radboud University, Nijmegen Medical Center, Nijmegen, Netherlands; 3 Shoklo Malaria Research Unit, Mahidol-Oxford Tropical Medicine Research Unit, Faculty of Tropical Medicine, Mahidol University, Mae Sot, Thailand; 4 Centre for Tropical Medicine and Global Health, Nuffield Department of Medicine, University of Oxford, Oxford, UK; 5 Lee Kong Chian School of Medicine, Nanyang Technological University, Singapore, Singapore; 6 School of Biological Sciences, Nanyang Technological University, Singapore, Singapore

**Keywords:** Diseases, Immunology, Microbiology

## Abstract

While vaccines are central to eradicate malaria, they remain elusive, with numerous malaria vaccine candidates showing limited efficacy in Phase II and III studies. Controlled human malaria infection studies have showed that human volunteers, immunized with *Plasmodium falciparum* sporozoites under drug cover, were protected experimentally from a subsequent challenge. Here, to identify new targets associated with protection, we utilized a previously developed screening approach, where we screened sera from protected and non-protected individuals against newly included hypothetical antigens in a *P. falciparum* antigen library. PfVFT1 was found to be associated with protection, with antibodies against PfVFT1 being detected in all protected individuals. We found that vaccine-induced mouse anti-PfVFT sera inhibited parasite reinvasion into RBCs, promoted complement deposition to induce parasite lysis, and supported phagocytosis and antibody-dependent cellular inhibition of the parasite. Together, these data indicate that PfVFT1-specific antibodies can engage multiple effector mechanisms relevant to antimalarial immunity.

## Introduction

Despite many decades of eradication efforts, malaria remains a major public health problem with high mortality and morbidity. With more than half of the world’s population at risk, there are 263 million clinical cases and 597 000 deaths in 2019^1^. Malaria is caused by the protozoan parasite *Plasmodium* and transmitted by *Anopheles* mosquitoes. The bulk of global burden is attributed to *P. falciparum*^1^. Early diagnosis, together with the use of insecticide-treated bed nets and combination drug therapies, has helped in reducing malaria mortality worldwide substantially. However, eradication efforts with only these control measures were not successful, mainly due to the emergence of drug-resistance parasites and insecticide-resistance vectors^2^. The development of new drugs and the development of efficacious and long-lasting vaccines for human use are critical if we are to attain the goal of global malaria eradication.

The complexity of the parasite life cycle has a profound impact on the vaccine design. Its life cycle alternates between the human and mosquito host. In the human host, *P. falciparum* exists in two distinct stages, the liver and the blood stages. Malaria vaccines have been developed to target the liver or the blood stage of the parasites in human. For both stages, vaccine development has taken various approaches. Cell-free approaches include the development of peptides, recombinant proteins, DNA plasmids, bacterial and viral vectors. The first approved malaria vaccine, RTS,S/AS01, also known as Mosquirix, was recommended by the World Health Organization (WHO) for use in children at risk of *P. falciparum* malaria in 2021. Subsequently, the WHO also recommended the use of R21/Matrix-M, another malaria vaccine. Both vaccines have the same antigen, which is a chimeric molecule based on the circumsporozoite protein (CSP), a major surface protein of the sporozoite, fused to the S antigen of the hepatitis B virus, formulated with potent adjuvants, AS01 and matrix M, respectively. Phase III clinical trials have shown that RTS,S/AS01’s efficacy was at best ~50% against clinical disease^3,4^. R21/Matrix-M boasts higher efficacy of up to 75% in seasonal sites, however the observed vaccine efficacy is significantly lower in older children and in standard sites^5^. In addition to the cell-free approaches, purified parasite preparations have also been developed as immunogens. It includes genetically-attenuated parasites, irradiated parasites, chemically-attenuated parasites, and live parasites under drug prophylaxis. More recently, whole parasite-based approaches have shown high efficacy^6–10^. This is likely attributed to the broader antigenic repertoire to which the host immune system is exposed^11–13^, as it allows for a full liver stage development and a partial blood stage development.

In our earlier study, we have shown sterile protection against a *P. falciparum* sporozoite challenge in volunteers immunized with live sporozoites under chloroquine prophylaxis^7^. In a subsequent study to explore the role of antibodies in the protection mediated in the same trial, we found that antibodies from protected individuals recognized a broad antigenic repertoire by screening the sera from the protected and non-protected individuals against a *P. falciparum* antigen library^14^. We identified PfTRAP, PfSEA-1 and PfMAEBL to be associated with protection and validated PfMAEBL by showing that anti-PfMAEBL antibodies can block liver stage development in human hepatocytes in vitro.

Here, we followed up our previous work to identify new antigens associated with protection. We expanded our *P. falciparum* antigen library to include 10 hypothetical antigens with unknown functions. Using the screening approach previously described^14^, we screened the sera from the protected and non-protected individuals from the trial by Roestenberg et al. against the 10 newly included hypothetical antigens. In this study, we unveil an antigen associated with protection. We also examine the potential of the antigen as a vaccine candidate by examining the mechanisms through which antibodies against the antigen, following vaccination, could mediate protection against *P. falciparum*.

## Results

### PfVFT1 found to be associated with protection

**Fig. 1:**
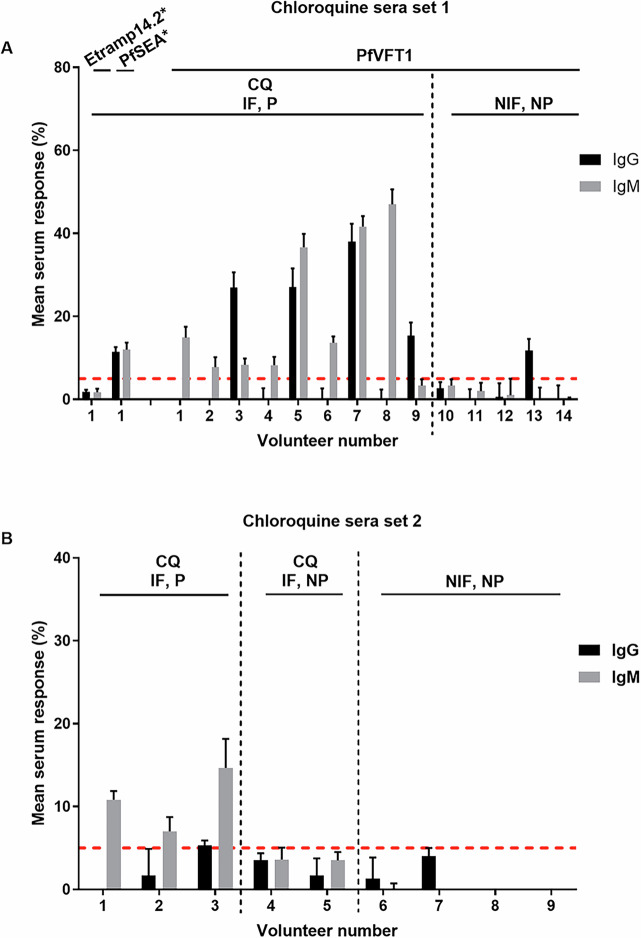
Serum response against PfVFT1. For both chloroquine sera set 1 (**A**) and 2 (**B**), each individual serum was incubated with transfected cells that expressed PfVFT1 on their surface. The population of cells that was bound by antibodies was determined *via* flow cytometry. Serum response above 5% was defined as positive serum response, indicated by the red dotted horizontal line. Serum was analysed in three independent experiments, with the mean antibody response being plotted. Error bar represents standard deviation. Etramp14.2-transfected cells served as negative control, while PfSEA-transfected cells served as positive control, as previously reported for chloroquine sera set 1^14^. For serum reactivity against *P. falciparum* antigens, comparisons of proportions between different groups of sera were performed using Fisher’s exact test on contingency table, where *p* value < 0.05 was considered significant. CQ chloroquine, IF infective bites, NIF non-infective bites, P protected, NP non-protected.

Using differential groups of sera from protected and non-protected individuals, we have previously reported a screening approach to identify antigens associated with protection, utilising our *P. falciparum* antigen library^14^. Here, we extended the study to look at 10 hypothetical antigens, newly included in the antigen library. The 10 hypothetical antigens were chosen based on its potential association with protection or its expression in the sporozoite stage in published literature^15–19^ (Supplementary Table 2). We first screened the same sera set^14^ (termed as chloroquine sera set 1) against the newly included 10 antigens (Supplementary Fig. 1a, b). PfVFT1 (PF3D7_0606800; previous gene IDs: MAL6P1.71, PFF0335C), numbered as PfL142 in our *P. falciparum* antigen library, was the only antigen found to be associated with protection—antibodies against PfVFT1, specifically IgM, were detected in all protected individuals (Fig. 1A, Supplementary Fig. 2). While IgG against PfVFT1 were detected in only 4 out of 9 of the protected individuals, IgM against PfVFT1 were detected in all the nine protected individuals. While the proportion of IgG responders is not statistically higher for PfVFT1 in protected individuals, the proportion of IgM responders against PfVFT1 is statistically higher in protected individuals (*p* = 0.023; Fig. 1A). We have previously found reactivity against PfSEA1 (PF3D7_1021800; previous gene ID: PF10_0212) and no reactivity against Etramp14.2 (PF3D7_1476100; previous gene ID: PF14_0729) in an earlier study^14^ and have included PfSEA1 and Etramp14.2 in this screen as positive and negative control (Supplementary Fig. 2a, b). While antibodies against PfVFT1 were also found in one of the non-protected individuals (#13), antibodies against the negative control, Etramp14.2, were also detected in individual #13—it is likely that this individual had a greater background noise. We followed up by examining the PfVFT1-transfected cells with sera from a subsequent study^20^, from which the chloroquine sera set was used (termed as chloroquine sera set 2). In this subsequent serum set, we also analysed the antibody responses against 10 antigens (Supplementary Fig. 1c, d) and detected antibodies against PfVFT1 in all protected individuals (*n* = 3) (*p* = 0.012; Fig. 1B, Supplementary Fig. 3). We have also included PfSEA1 and Etramp14.2 in this screen as positive and negative control. (Supplementary Fig. 2c, d).

### PfVFT1 expressed on blood stage parasites

Until date, PfVFT1 is a protein with unknown function. This is likely because the only human malaria it exists in is *P. falciparum*. With the exception of *P. reichenowi* and *P. gaboni*, it is not found in any other *Plasmodium* species. We first determined the stages during which the PfVFT1 antigen was expressed by performing immunofluorescence studies on liver sporozoites and blood stage parasites. To this end, we first cloned and expressed PfVFT1 as a secreted antigen. After immunization of mice with the purified PfVFT1, we obtained mouse anti-PfVFT1 sera. PfVFT1 specificity of the sera was verified by western blot using purified PfVFT1 (Supplementary Fig. 4a), ELISA using purified PfVFT1-coated plates (Supplementary Fig. 4b), FACS using PfVFT1-transfected cells (Supplementary Fig. 4c), ELISA using *P. falciparum* 3D7 schizonts lysate (Supplementary Fig. 4d), and western blot using *P. falciparum* 3D7 schizonts lysate (Supplementary Fig. 6c). The PfVFT1-specific antibody responses in the mouse anti-PfVFT1 sera were also found to be high (Supplementary Fig. 4b–d).

**Fig. 2:**
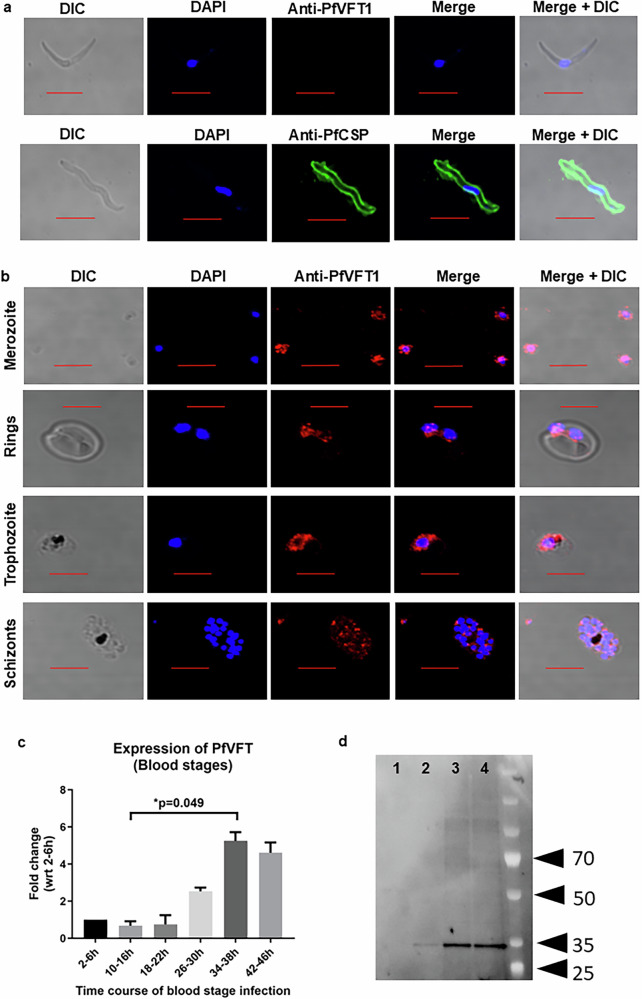
PfVFT1 antigen staining on parasites. **a** Liver sporozoite slides were fixed, stained with either anti-PfVFT1 or anti-CSP (PF3D7_0304600; previous gene IDs: MAL3P2.11, PFC0210C) sera. Red bar represents 5 µm. Representative images from three independent experiments shown. **b** Blood stages parasite slides were prepared for the merozoite, ring, trophozoite and schizont stage. The slides were fixed and stained with pooled mouse anti-PfVFT1 sera. Red bar represents 5 µm. Representative images from three independent experiments are shown. **c** Samples were collected at 2–6, 10–14, 18–22, 26–30, 34–38, 42–46 h of the blood cycle. PfVFT1 RNA expression at the various time points was examined using qPCR. Expression was analysed in three independent experiments, and the mean fold change (with respect to 2–6 h) was plotted. Comparisons between the mean fold changes at different timepoints were analysed using Friedman test, corrected with Dunn’s multiple comparison test. **d** Samples were collected at 10–14, 26–30 and 42–46 h of the blood cycle. PfVFT1 protein expression was examined using specific western blot. Representative blot from two independent experiments is shown. 1: culture supernatant; 2: ring stage (10–14 h); 3: trophozoite stage (26–30 h); 4: schizont stage (42–46 h).

**Fig. 3:**
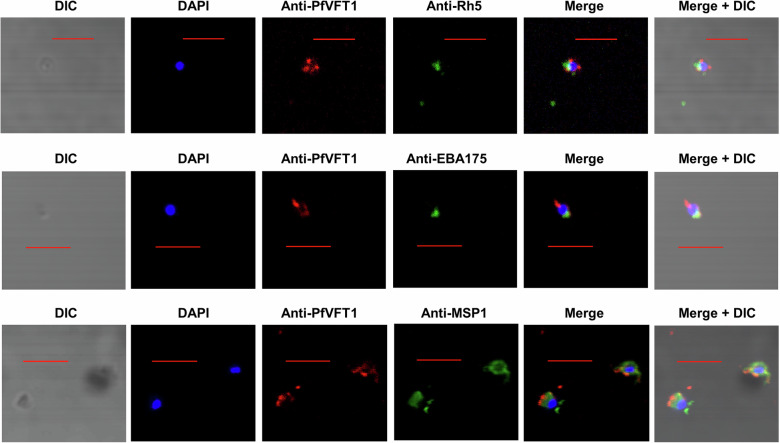
PfVFT1 antigen did not co-localise with rhoptry protein Rh5, micronemal protein EBA175 and merozoite surface protein MSP1. Merozoite slides were fixed and stained with pooled mouse anti-PfVFT1 sera (indicated by the green signal), in addition to one of the following sera, Rh5, EBA175 or MSP1 (indicated by the red signal). Red bar represents 5 µm. Representative images from three independent experiments are shown.

Using the anti-PfVFT1 sera, immunofluorescence studies showed that PfVFT1 was not expressed by liver sporozoites (Fig. 2a), but was expressed by all stages of the blood stage parasites, ring, trophozoite, schizont and merozoite (Fig. 2b, Supplementary Fig. 5a). We also detected positive binding by the mouse anti-PfVFT1 sera in blood stage parasite ELISA (Supplementary Fig. 4d). We went on to examine the PfVFT1 RNA expression throughout the blood stages. We found that PfVFT1 mRNA is expressed at higher levels at the later stages such as trophozoites (34–38 h), compared to the ring stage (10–14 h) (*p* = 0.049; Fig. 2c). The PfVFT1 protein were also detected at higher levels at the trophozoite (26–30 h) and schizonts (42–46 h) stages, compared to the ring stage (10–14 h) (Fig. 2d). We did not detect the PfVFT1 protein in the culture supernatant (Fig. 2d). To determine the subcellular localisation of PfVFT1 in the parasite, we looked at three subcellular locations: rhoptry, microneme and merozoite surface. PfVFT1 did not co-localise with the rhoptry protein, Rh5 (PF3D7_0424100; previous gene IDs: MAL4P1.224, PFD1145C) and micronemal protein, EBA-175 (PF3D7_0731500; previous gene IDs: MAL7P1.176, PF07_0128) (Fig. 3, Supplementary Fig. 5b). While PfVFT1 did not exhibit a complete co-localisation with the merozoite surface protein, MSP1 (PF3D7_0930300; previous gene IDs: PFI1475W) (Fig. 3, Supplementary Fig. 5b), there seemed to be pockets of co-localisation between the two proteins. It is possible that PfVFT1 is surface-localised like MSP1, but may not have the same localisation pattern as MSP1. Previous study using fractionation of detergent-resistance membranes showed that PfVFT1 clusters with GPI-anchored parasite proteins such as MSP1^21^.

### Anti-PfVFT1 serum inhibits P. falciparum invasion in RBCs

**Fig. 4:**
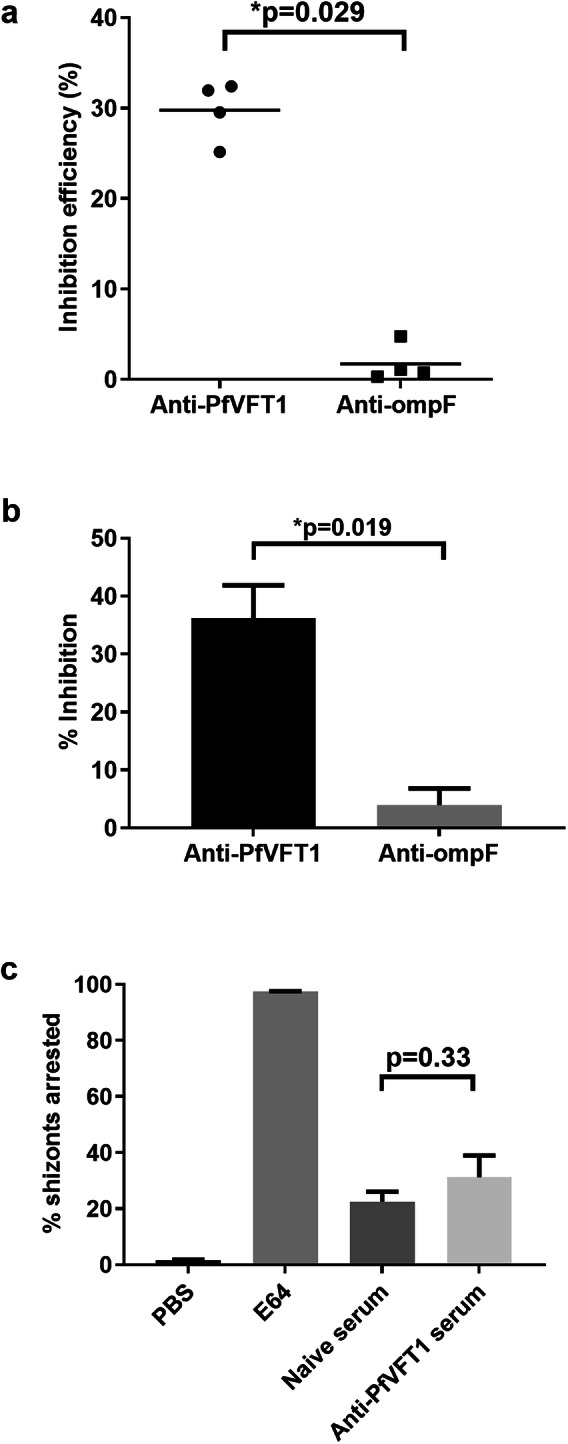
PfVFT1 inhibited *P. falciparum* invasion into RBCs. **a** *P. falciparum* schizont reinvasion assays. Each of the three independent experiments, with a final 1% parasitemia, 2% hematocrit and 1:20 serum dilution (either pooled mouse naïve sera, pooled anti-PfVFT1 sera or pooled anti-ompF sera). Inhibition efficiency was defined as the ratio of the subtraction of parasitemia in the test well from the parasitemia in the control well to the parasitemia in the control well, expressed as a percentage. Values above 0% indicated positive inhibition (no invasion) while values below 0% indicated no inhibition (positive invasion). Mean reinvasion inhibition efficiency of the three independent replicates was plotted, with each dot representing each isolate. Bars indicate mean reinvasion inhibition efficiency for the four isolates. The four isolates used are clinical isolates from Maesot, Thailand. Unpaired t-tests were used to compare groups. **b** *P. falciparum* 3D7 merozoite reinvasion assays were set up with a merozoites: RBCs ratio of 5:1 and a final haematocrit of 2%. Mean values from three independent experiments were plotted (three technical experiment repeats), with error bars indicating standard deviation. Unpaired t-tests were used to compare groups. **c** *P. falciparum* 3D7 schizont arrest assays were set up with a final 3% parasitemia, 2% haematocrit and 1:20 serum dilution. The percentage of schizonts arrested at 12 h was calculated by the number of schizonts at 12 h divided by the number of schizonts at 0 h. For each well, 200 parasite-infected RBCs were counted. Mean values from three independent experiments (three technical experiment repeats) were plotted, with error bars indicating standard deviation. Mann-Whitney U tests were used to compare groups.

We hypothesised that the antigen might have a role in invasion in RBCs, as PfVFT1 was expressed in blood stage parasites, more specifically by merozoites. We first examined the capability of the sera from the vaccinees in both our cohorts to inhibit *P. falciparum* reinvasion into RBCs and found that the sera from both protected and non-protected vaccinees in both cohorts did not inhibit *P. falciparum* reinvasion (Supplementary Fig. 6). The concentrations of PfVFT antibodies in the sera may be too low and insufficient to mediate a functional effect (Supplementary Fig. 6). Using anti-PfVFT mouse sera with high antibody titres, the reinvasion assays showed that the mouse anti-PfVFT1 serum inhibited the invasion of four different clinical *P. falciparum* isolates (from Maesot, Thailand) into RBCs, with an efficiency of ∼30% (*p* = 0.029; Fig. 4a). While the anti-PfVFT1 serum inhibited parasite reinvasion in the schizont reinvasion assay, the inhibition could be due to an inhibition of merozoite invasion into RBCs and/or an inhibition of schizont rupture to release the merozoites for subsequent invasion. Hence, we repeated the invasion assay using merozoites. The merozoite invasion assay showed that the anti-PfVFT1 serum inhibited merozoite invasion into RBCs, with an efficiency of ∼35% (*p* = 0.019; Fig. 4b). Through schizont arrest assays, we found that the schizonts were not arrested in the presence of the anti-PfVFT1L serum (Fig. 4c)—there was no difference in the percentage of schizonts arrested with either the naïve or anti-PfVFT1 serum (*p* = 0.33). Taken together, the anti-PfVFT1-mediated reinvasion inhibition is likely due to an inhibition of merozoite invasion into RBCs, and not due to blockage of the schizont bursting to release the merozoites.

### PfVFT1 mutants have a growth defect

As PfVFT1 has a role to play in merozoite invasion into RBCs, we investigated the essentiality of the antigen. We took the CRISPR/Cas9 approach^22^ to disrupt the gene by homologous recombination. 3D7 blood stage parasites were transfected with the pUF1-Cas9 plasmid and the pL7-*PfVFT1* plasmid (Supplementary Fig. 7a). Our attempts were successful, generating 3D7^∆PfVFT1^ mutants. PCR analysis showed *PfVFT1* gene disruption and integration of the resistance cassette through a double-crossover recombination (Supplementary Fig. 7b). Western blot analysis confirmed the absence of PfVFT1 protein expression by the mutants (Supplementary Fig. 7c). These demonstrated that PfVFT1 is not an essential antigen needed for blood stage parasite survival.

**Fig. 5:**
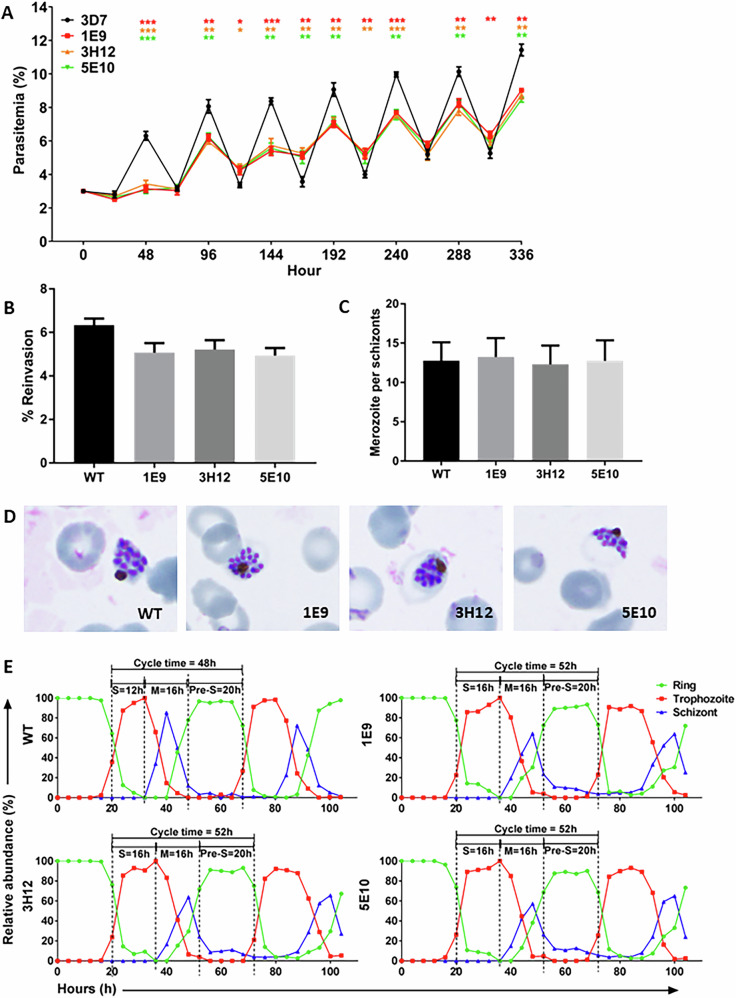
Erythrocytic growth of 3D7^∆PfVFT1^ mutants. The growth of three 3D7^∆PfVFT1^ clones (1E9, 3H12, 5E10) was compared with the parental 3D7. **A** Replication curves. Freshly thawed parasite cultures were set up with a final parasitemia of 3%. Every two days (ring stage), the cultures were diluted to a final parasitemia of 3%. Daily parasitemia was measured. For each smear, 1000 parasites were counted. Three independent experiments (three technical experiment repeats) were carried out, with the mean parasitemia being plotted. Error bars indicate standard deviation. Unpaired t test with Welch correction and false discovery rate adjustment was used to compare the parasitemia between the parental 3D7 and the three mutant clones. *P*-values: ***p-value < 0.001; **p-value < 0.01; *p-value < 0.05. **B** The merozoite reinvasion assay was set up with a merozoites: RBCs ratio of 5:1 and a final haematocrit of 2%. Mean values from three independent experiments (three technical experiment repeats) were plotted, with error bars indicating standard deviation. Mann Whitney U test was used to compare between isolates. **C** Merozoite count assay. Parasite cultures were set up with a final parasitemia of 3% and a final haematocrit of 3%, and followed till the schizont stage (42–46 h). Smears were then prepared after 12 h and the number of merozoite per schizont was counted. 200 schizonts were counted for each culture. Mean values from three independent experiments (three technical experiment repeats) were plotted, with error bars indicating standard deviation. Mann-Whitney U tests were used to compare groups. **D** Representative images of the parental 3D7 and the 3D7^∆PfVFT1^ mutants schizonts from three independent merozoite count assays. **E** Cell cycle analysis of the 3D7^∆PfVFT1^ mutants. Parasite cultures (a final parasitemia of 3% and a final haematocrit of 3%) were followed for 104 h. Smears were prepared every 4 h. For each smear, 1000 parasites were counted. The relative abundance of the ring, trophozoite and schizont stages was calculated. The cell cycle analysis was repeated in another independent experiment with a final parasitemia of either 3% or 0.2%, and a final haematocrit of 3% (Supplementary Fig. 7).

While PfVFT1 is not an essential antigen, we hypothesised that the disruption of *PfVFT1* might affect parasite growth in the mutants, given its implicated role in invasion into RBCs. We first studied the replication curves of 3D7^∆PfVFT1^ mutants. We found that, even when all parasite cultures were set up similarly to an initial parasitemia of 3%, there is a general trend of the final parasitemia of the 3D7^∆PfVFT1^ mutants being lower than the parental 3D7 after a blood cycle (i.e. at 48 h, 96 h, 144 h, 192 h, 240 h, 288 h, 336 h, which corresponds to day 2, 4, 6, 8, 10, 12, 14 respectively) (Fig. 5A). At 48 h, the parasitemia of the 3D7^∆PfVFT1^ mutants was significantly lower than the parental 3D7.

The lower parasitemia of the 3D7^∆PfVFT1^ mutants was not due to an impairment of invasion into RBCs or an impairment of merozoite development. Using merozoite invasion assay, while we observed a consistently lower reinvasion into the RBCs with the 3D7^∆PfVFT1^ mutants than the parental 3D7 (Fig. 5B), it was not significant. It may be due to the redundancy of the various invasion pathways of the *P. falciparum* parasite. We also counted the merozoites per schizont and compared the 3D7^∆PfVFT1^ mutants with the parental 3D7. There was no significant difference in the number of merozoites per schizont (Fig. 5C, D), indicating no impairment of merozoite development.

Further cell cycle analysis showed that the 3D7^∆PfVFT1^ mutants do have a growth defect. We followed freshly thawed parasite cultures for 104 h and collected samples every 4 h. The relative abundance of the ring, trophozoite and schizont stages was calculated at each time point. We found that the 3D7^∆PfVFT1^ mutants have a longer cell cycle of 52 h, as compared to the 48 h cell cycle of the parental 3D7 (Fig. 5E and Supplementary Fig. 8). This seemed to stem from a longer trophozoite S phase, resulting in a later entry into the schizont M phase. PfVFT1 could regulate the transition from the trophozoite phase to the schizont phase. We complemented the 3D7^∆PfVFT1^ 1E9 mutant through episomal insertion (Supplementary Fig. 9) and found that the gene complementation rescued the growth defect (Supplementary Fig. 8). Complemented 3D7^∆PfVFT1^ 1E9 mutant was observed to have cell cycle of 48 h, similar to the parental 3D7.

### Anti-PfVFT1 serum mediated parasite phagocytosis and antibody-dependent cellular inhibition by macrophages

**Fig. 6:**
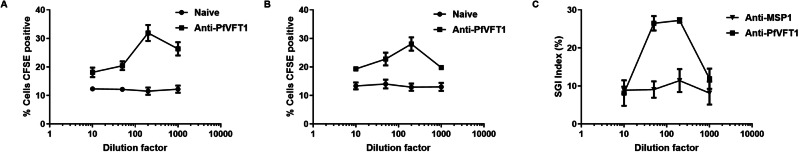
Anti-PfVFT1 serum mediated merozoite and iRBC phagocytosis, and ADCI by macrophages. Phagocytosis assays were performed, with either (**A**) iRBCs (schizonts) or (**B**) merozoites and RAW264.7 mouse macrophage as host cells. The CFSE-stained iRBCs (schizonts) or merozoites were opsonized with diluted sera (1:10, 1:50, 1:200, 1:1000), where sera are either pooled naïve mouse sera or pooled mouse anti-PfVFT1 sera. The opsonized CFSE-stained iRBCs were added to the RAW264.7 cells at a iRBC: RAW264.7 ratio of 25:1. For merozoites, the merozoite: RAW264.7 ratio is 10:1. Phagocytosis was defined as the percentage of CFSE-positive RAW264.7 cells (RAW264.7 cells with internalised CFSE-stained iRBCs or merozoites). Mean values from three independent experiments (three technical experiment repeats) were plotted, with error bars indicating standard deviation. Mann-Whitney U tests were used to compare groups. **C** ADCI assay was set up with RAW264.7 macrophages, MACS-sorted schizonts and diluted sera (where sera are either pooled mouse anti-MSP1 sera or pooled mouse anti-PfVFT1 sera) at 0.5% parasitemia and 2% haematocrit. At 86 h, the trophozoites in the media were stained with Hoechst and FITC-conjugated anti-mouse CD45. Parasitemia was defined as Hoechst-positive and FITC-negative events. The specific growth inhibitory index (SGI) was calculated as the percentage inhibition relative to that for the control sera. Mean values from three independent experiments (three technical experiment repeats) were plotted, with error bars indicating standard deviation. Paired t-tests were used to compare groups at the serum dilutions.

In order to have a better understanding of the potential of PfVFT1 as a vaccine candidate, we set out to investigate the functionality of the antibody induced following PfVFT1 immunization in mice. We examined if the mouse anti-PfVFT1 serum could facilitate the effector mechanisms of phagocytes. To this end, we utilized the mouse anti-PfVFT1 serum and investigated if the mouse anti-PfVFT1 serum could facilitate the cell-dependent effector mechanisms such as phagocytosis, using corresponding mouse macrophage cell line with compatible Fc receptors, RAW264.7. First, we found that the anti-PfVFT1 serum facilitated the phagocytosis of iRBCs (Fig. 6A; *p* = 0.029) and merozoites (Fig. 6B; *p* = 0.028) by RAW264.7. In the presence of the anti-PfVFT1 serum, mouse macrophages RAW264.7 were also able to induce antibody-dependent cellular inhibition (ADCI) and inhibited parasite growth, as indicated by a higher specific growth inhibitory index at serum dilution factor of 50 (*p* = 0.013) and 200 (*p* = 0.019) (Fig. 6C). The lack of dose-dependent effect at lower antibody dilution could be due to prozone effect^23^, where loss of activity was observed at high antibody concentrations. The prozone effect has been reported, thus far, in in vitro immune assays^24^. Phagocytosis of *Salmonella* has been reported to be impaired at high antibody concentrations^25^, while one of the causes of false-negative HRP-2 RDTs in samples with hyperparasitaemia has been attributed to high antibody levels (the prozone effect)^26,27^. Similar cases have been reported in in vitro immune testing for HIV^28^, secondary syphilis^29^ and COVID-19^30^. Despite reports of prozone effect reported in COVID-19 immunoassay testing^30^, the high antibody titres following COVID-19 are functional and are still found to be associated with protective efficacy^31,32^. Hence, it is possible that the prozone effect is an artefact in in vitro immunoassay testing. Despite multiple reports of the prozone effect in in vitro immune assays, the exact mechanism involved remains unknown and warrants further investigation. To study if the anti-PfVFT1 serum can facilitate antibody-dependent respiratory burst, we isolated mouse neutrophils. Using in a previously described respiratory burst assay^33^, we found that the neutrophils did not induce significant respiratory burst in the presence of the anti-PfVFT1 serum (Supplementary Fig. 10). The absence of downstream response in the neutrophils differs from that observed with the phagocytosis and ADCI in RAW264.7 macrophages. The differential responses of the RAW264.7 macrophages and neutrophils likely reflect fundamental differences in Fc receptor expression and activation thresholds between these cell types^34^. It may be worth noting that there is a slight increase in respiratory burst with the pooled anti-PfVFT sera-opsonised parasites around the 5 min mark, which was absent with the pooled naïve sera-opsonised parasites (Supplementary Fig. 10). PMA crosses the plasma membrane without the aid of a membrane receptor and directly activates protein kinase C by translocation of the cytosolic isoforms to the cell membrane^35^. Unlike PMA stimulation, respiratory burst mediated by specific antibodies requires membrane receptor engagement. It may be possible that the neutrophils need other priming factors, such as complement or cytokines for efficient response via receptor engagement^36^.

### Anti-PfVFT1 serum mediated complement deposition and merozoite lysis

Our data suggested that the effects of PfVFT1 were mainly implicated in the blood stage. As complement is one of the prominent host defence mechanisms in the blood compartment, we investigated if the mouse anti-PfVFT1 serum could mediate complement deposition to activate the classical complement pathway, leading to parasite lysis.

**Fig. 7:**
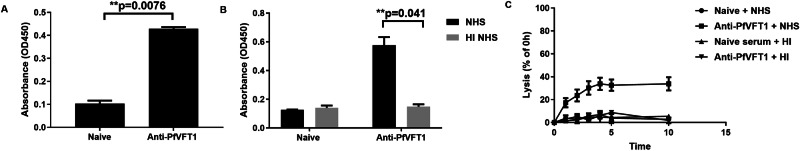
Anti-PfVFT1 serum mediated complement deposition and merozoite lysis. C1q (**A**) and C5-C9 (**B**) deposition were measured by ELISA. 96well plates were coated with merozoites at 5 × 10^6^ merozoite/well. For C1q deposition ELISA, recombinant C1q was used. For C5-C9 deposition, NHS or HI NHS was used. Mean values from three independent experiments (three technical experiment repeats) were plotted, with error bars indicating standard deviation. Paired t-test was used to compare groups. **C** Merozoite lysis assay. Merozoites were added into 96-well plates at 5 × 10^6^ merozoite/well, in triplicate, and incubated with sera (either pooled naïve mouse or pooled mouse anti-PfVFT1, diluted 1:20) and either 25% NHS or HI NHS. Mean values from three independent experiments (three technical experiment repeats) were plotted, with error bars indicating standard deviation. Kruskal–Wallis test was used to compare groups.

Using ELISA, we first looked at the deposition of C1q and C5-C9 on the parasite. In the presence of anti-PfVFT1 serum, there were higher levels of C1q deposition (*p* = 0.0076) on the parasite (Fig. 7A). Similarly, higher levels of C5-C9 deposition (*p* = 0.041; Fig. 7B) were observed in the presence of anti-PfVFT1 serum and normal human serum (NHS). We then went on to examine merozoite lysis. In the presence of the anti-PfVFT1 serum, we observed merozoite lysis as early as 1 min into the assay and a maximum of ∼35% of the merozoites being lysed by 5 min (*p* = 0.04; Fig. 7C). No merozoite lysis was observed in the absence of the PfVFT1-specific serum or when the NHS was heat-inactivated. The merozoite lysis observed in the presence of anti-PfVFT1 serum was likely to be attributed to the higher levels of complement deposition, suggesting that the anti-PfVFT1 serum could mediate complement deposition to activate the classical complement pathway, leading to lysis.

### Genetic variation of PfVFT1

Our data have suggested the potential of PfVFT1 as a vaccine candidate. Hence, we investigated the genetic variation of the antigen to have a preliminary indication of the potential vaccine coverage. We first explored the data provided by PlasmoDB, where an alignment of 202 isolates revealed no non-coding SNPs, seven synonymous SNPs and four non-synonymous SNPs (Supplementary Fig. 11a). The four non-synonymous SNPs were shown in Supplementary Fig. 11b. We also sequenced 90 *P. falciparum* field isolates from Maesot, Thailand, and did not detect any non-coding SNPs and non-synonymous SNPs (Supplementary Fig. 11c). Only one synonymous SNP was found. Taken together, PfVFT1 is a highly conserved antigen.

## Discussion

Global malaria eradication requires a concerted effort that involves early disease diagnosis, the use of disease control measures such as insecticide-treated bed nets, and the development and implementation of new drug therapies and vaccines. The seemingly inevitable emergence of insecticide and drug resistance stressed the need for a malaria vaccine. The approval of the RTS,S/AS01 and R21/Matrix-M malaria vaccines represents a significant step towards global malaria eradication. However, the vaccine efficacy of RTS,S/AS01 was suboptimal and insufficient, and more studies are needed to understand the lower vaccine efficacy of R21/Matrix-M in older children. Hence, it is important to identify new vaccine candidates.

One key finding of this study is that, using the sera set previously described^14^ that contained sera from protected and non-protected volunteers which were exposed to infective mosquito bites under chloroquine prophylaxis, our screen against the 10 newly included hypothetical antigens in our antigen library identified PfVFT1 to be associated with protection. We further verified this with a second sera set obtained from another group of volunteers immunised following the same protocol^20^, where we also found antibodies against PfVFT1 in all protected individuals. More specifically, IgM against PfVFT1 was detected in all protected individuals in both sera set, suggesting a potential importance of IgM in protection. This is in line with a study by Arama et al., where the authors found that resistance to malaria in African population is associated with a greater IgM response^37^. Similarly, Boyle et al. found robust and long-lived IgM responses, specifically merozoite-specific IgM, during malaria in children and adults with lifetime exposure^38^. Our finding, that PfVFT1 is associated with protection, is in agreement with another study, using a distinct antigen library, that detected PfVFT antibodies in individuals living in endemic areas with regular exposure to infection with *P. falciparum* parasites and identified PfVFT1 to be associated with protection against clinical malaria in a cohort of Kenyan children^16^. Until now, PfVFT1 has not been well-characterized, and its function is unknown. It is a parasite-specific antigen, and the only human *Plasmodium* species that expresses PfVFT1 is *P. falciparum*^39^. It is a relatively small protein of ∼35 kDa, with a signal peptide. No identifiable membrane anchor has been detected in the protein through bioinformatics analysis^40^. While it has been detected in the membrane fractions of *P. falciparum*^21^, it was not likely GPI-anchored^41^. Hence, it is likely that it associates itself with a membrane protein to enable its localisation to the membranes.

Using immunofluorescence, we found that PfVFT1 is not expressed in sporozoites, but in all stages of the blood stages. While PfVFT1 RNA expression was the highest at the later phases of the blood stages, it was also expressed on merozoites and ring stage parasites. This is not surprising since our immunization regime did expose the host immune system to blood stage parasites. We have detected parasites in the peripheral blood between day 6–9 following each of the three immunizations^7^, confirming the exposure to blood stage parasites. Hence, it is likely that, even with the relatively short exposure to blood stage parasites, the host immune system was able to develop antibodies against PfVFT1. It is worth noting that we did not determine the RNA expression of PfVFT1 in *P. falciparum* liver stages. It is possible that PfVFT1 might also be expressed in liver merozoites, and it could possibly be a target for inhibitory antibodies.

Attempts to disrupt the *PfVFT1* gene by the CRISPR-Cas9 system have been successful in blood stage parasites. While PfVFT1 is not essential for the survival of blood stage parasites, the 3D7^∆PfVFT1^ mutants have a slight growth defect. With a longer trophozoite S phase and a later entry into the schizont M phase, PfVFT1 could regulate the transition from trophozoite to schizont. Various antigens have been implicated in the *P. falciparum* invasion, namely EBA175, EBA140, EBA181, PfRh1, PfRh2a, PfRh2b, and PfRh4^42–46^. Similarly, attempts to disrupt some of these genes have been successful^44,47^. These highlighted the considerable redundancy in *P. falciparum* invasion pathways.

Having found that PfVFT1 was expressed by blood stage parasites and was associated with protection in two different controlled human malaria infection studies^7,20^, this suggested that the protection observed might be mediated by immune mechanisms against this antigen. This was surprising since it was previously demonstrated that this immunization protocol induced protection mainly against the pre-erythrocytic stage of the infection in humans^48^. However, mouse studies investigating the same immunization protocol have shown that full sterile protection was due to defences against both the pre-erythrocytic and blood stages^49^. It must be stressed that, due to ethical reasons, it is not possible to let the blood stage infection fully develop in the human volunteers, and thus any inhibitory effect on the blood stages might have been missed.

We investigated whether the mechanisms through which antibodies against PfVFT1, following vaccination, could mediate protection against *P. falciparum*. Different functional assays were tested, and we found that vaccine-induced mouse antibodies against PfVFT1 could inhibit *P. falciparum* reinvasion into RBCs, mediate phagocytosis of infected RBCs and merozoites and ADCI by macrophages. It was also worth noting that the ability of the mouse anti-PfVFT antibodies to mediate phagocytosis of infected erythrocytes was unexpected, as we did not observe PfVFT on the surface of infected erythrocytes. It is possible that the infected erythrocytes might have ruptured to release merozoites during opsonisation and it may be the released merozoites being opsonised and eventually phagocytosed. Although anti-PfVFT1 antibodies did not induce a respiratory burst in neutrophils, these data are promising. Anti-merozoite opsonizing antibodies mediating phagocytosis assays have been associated with protection against clinical disease^50^, and antibodies mediating ADCI have also been correlated with protective immunity^51^.

In addition, antibodies against PfVFT1 supported the deposition of complement components, C1q and C5-C9, leading to merozoite lysis. While it was reported that the parasite evades complement pathway, where the merozoites recruit complement regulators such as factor H^52^, and the infected erythrocytes binds to non-specific IgM to limit binding by parasite-specific IgG to prevent lysis^53^, it has also been demonstrated that C1q binding to merozoites, in a complement fixation assay, was associated with protective immunity against malaria^54^. The rapid series of events that follows upon complement activation to parasite lysis occurred within a few minutes, which could potentially have an impact on limiting parasite growth.

It is worth noting that one key limitation of this study is the utilisation of sera from mice immunised with PfVFT antigen-vaccine formulated with Complete Freund’s adjuvant, which is historically of limited value when used to predict vaccine-induced immune responses in human subjects. While this study was intended primarily as a proof-of-concept to evaluate the immunogenicity of the antigen and to characterize the nature of the induced antibody responses under strong immunostimulatory conditions, further evaluation with more clinically relevant adjuvants or vaccine platforms would be necessary to better understand the potential vaccine-induced immune responses in human. Additionally, one other limitation is the limited amount of the human serum samples from the human challenge to perform purification of anti-PfVFT antibodies from the protected human individuals for the downstream assay analysis. The purification of anti-PfVFT antibodies from the protected human individuals for the downstream assay analysis would provide stronger evidence of the causal relationship between anti-PfVFT antibodies and protection in the challenge model.

In conclusion, vaccine-induced mouse antibodies against PfVFT1 could inhibit parasite reinvasion into RBCs, mediate complement deposition to induce parasite lysis and mediate phagocyte functions such as phagocytosis and antibody-dependent cellular inhibition of the parasite. Further studies to investigate the exact role of PfVFT1 in parasite invasion and to evaluate vaccine-induced responses following more with more clinically relevant adjuvants or vaccine platforms, particularly in human, are needed to establish the casual relationship of anti-PfVFT antibodies and protection. While the ability to inhibit parasite reinvasion into RBCs may be modest, anti-PfVFT1 antibody can mediate complement deposition to induce parasite lysis and mediate phagocytosis and antibody-dependent cellular inhibition of the parasite. Collectively, vaccine-induced antibodies against PfVFT1 are functional and may mediate effector mechanisms relevant to antimalarial immunity Our genetic analysis, coupled with efforts by Plasmodb^39^, showed that PfVFT1 is a highly conserved. Taken together, PfVFT1 may be considered as a potential vaccine candidate for further investigation, that could be included in multi-component vaccine approaches against malaria.

## Methods

### Study sera

Sera were obtained from two previous clinical trials carried out at the Radboud University Nijmegen Medical Center (Nijmegen, Netherlands), in accordance with principles of good clinical practice and with prior approval from the Central Committee for Research Involving Human Subjects of The Netherlands (NCT00442377 and NCT01422954). The trial (NCT00442377) was approved by the institutional review board at the Radboud University Nijmegen Medical Centre. The Central Committee for Research Involving Human Subjects of the Netherlands approved the trial (NCT01422954). Written informed consent was obtained from all study participants in accordance with the Declaration of Helsinki for Human Research. For chloroquine sera set 1 (NCT00442377), study subjects (*n* = 14) were exposed to either infective (*n* = 9) or non-infective (*n* = 5) mosquito bites with concurrent chloroquine prophylaxis^7^. Drug prophylaxis was given from day 0-90 while exposure to infective or non-infective bites was performed on day 7, 35, 63. Challenge was performed 28 days after the end of drug prophylaxis on day 118. For chloroquine sera set 2 (NCT01422954), study subjects (*n* = 9) were exposed to either infective (*n* = 5) or non-infective (*n* = 4) mosquito bites with concurrent chloroquine prophylaxis^55^. Drug prophylaxis was given from day 0-105 while exposure to bites was performed on day 22, 50, 78. Challenge was performed 112 days after the end of drug prophylaxis on day 218. For both clinical trials, serum samples were taken at two time points: the day before the first immunization and the day before challenge.

### Plasmodium culture

*P. falciparum* NF54 strain was used in the clinical trials^7,20^. A laboratory clone 3D7 (a cloned line derived from NF54) was used for the *P. falciparum* antigen library construction, and the in vitro assays. For clinical isolates, the isolates were collected from Thai patients under the ethical guidelines in approved protocols; OXTREC 027-025 (University of Oxford, Centre for Clinical Vaccinology and Tropical Medicine, UK) and MUTM 2008-215 from Ethic committee of Faculty of Tropical Medicine of Mahidol University. The isolates were collected between in 2009 and 2010 from malaria patients, attending the Shoklo Malaria Research Unit clinics, Mae Sot, Thailand, with no prior antimalarial therapy and with microscopically-confirmed *P. falciparum*.

Blood stage parasites were cultured in vitro using RPMI-HEPES medium pH 7.4 supplemented with 50 μg/ml hypoxanthine, 25 mM NaHCO_3_, 2.5 μg/ml gentamicin and 10% serum at 37 °C, 5% CO_2_. For liver sporozoites, salivary glands from infected *Anopheles stephensi* mosquitoes were hand-dissected, collected in complete William’s B culture medium without serum, and homogenized in a homemade glass grinder to harvest the sporozoites.

### *P. falciparum* antigen library

The *P. falciparum* antigen library was as previously described^14^. Briefly, nucleotide sequences encoding for *P. falciparum* antigens were amplified *via* PCR, using either 3D7 genomic DNA or RNA as template, and cloned into the pDisplay vector (Invitrogen). The resultant plasmids were then transfected into HEK293 cells using lipofectamine 2000 (Invitrogen) for surface expression of the antigens. The antigen has a hemagglutinin (HA) tag at the N-terminal of the antigen and a myc tag at the C-terminal of the antigen, allowing detection of the antigen using an anti-HA (Sigma) or anti-myc (Miltenyi Biotec) antibodies.

### Antibody profiling of patients’ serum response

Determination of the patients’ antibody profile was as previously described^14^. Transfected cells, expressing *P. falciparum* antigens on the cell surface, were first incubated with human serum (diluted 1:100 in 10% FBS (in PBS)) from the clinical trials (NCT00442377 and NCT01422954). The cells were then incubated with a double stain, consisting of Alexa Fluor 488-coupled secondary antibodies (Invitrogen; diluted 1:500) and propidium iodide (PI; diluted 1:2500). Cells were read on Accuri C6 (BD Biosciences) and analyzed using FlowJo (Tree Star). Parallel to the determination of presence of specific antibodies in the patients’ sera, the transfected cells were also stained separately with anti-myc or anti-HA antibodies (diluted 1:100) to determine transfection efficiency. Serum was analysed in three independent experiments, with the mean antibody response being plotted.

The analysis followed the below four steps: (1) determining the transfection efficiency of the transfected cells: the proportion of cells that were transfected and expressing the *P. falciparum* antigen, which was defined as Alexa Fluor 488-positive and PI-negative labelling (PI-negative labelling indicates live cells) (Fig. S1A, Gate 3); (2) determining the presence of *P. falciparum*-specific antibody response in each serum (using the pre-immune sera for baseline gating) in sera set 1 (Fig. S1B) and sera set 2 (Fig. S1C) and was defined by Alexa Fluor 488-positive and PI-negative labelling (Gate 3), where antibody response in pre-immune sera was subtracted from the antibody response in post immunisation sera; (3) quantifying the *P. falciparum*-specific antibody reactivity by normalizing the antibody response to transfection efficiency: the proportion of cells with bound sera antibodies (from step 2) was divided by the transfection efficiency (from step 1), and expressed as a percentage; (4) determining if the *P. falciparum*-specific antibody response was positive: *P. falciparum*-specific antibody response was defined as positive when the antibody response (from step 3) was above 5% (i.e., 5% over the baseline pre-immune sera).

### Generation of recombinant PfVFT1 protein and mouse anti-PfVFT1 sera

To generate the recombinant PfVFT1 protein, full length codon-optimised *PfVFT1* gene (PF3D7_0606800; previous gene IDs: MAL6P1.71, PFF0335C) was amplified using primer pair rPfVFT1cdF and rPfVFT1cdF (Supplementary Table 1) and then cloned into expression vector, p3XFLAG-CMV-9 (Thermo Fisher Scientific), between HindII and BamHI restriction enzyme sites. The resultant vector was transiently transfected into HEK293 cells, using lipofectamine 2000, to allow expression of secreted PfVFT1 with three FLAG epitopes at its N-terminal. Using anti-FLAG M2 affinity matrix (Sigma-Aldrich), PfVFT1 antigen was purified from the spent culture media *via* the FLAG tag, according to manufacturer’s protocol. HEK293 cells were cultured in DMEM (HyClone) supplemented with 10% FCS (GIBCO) and 1% penicillin–streptomycin solution (100X, stock solution, GIBCO).

To generate mouse anti-PfVFT1 sera, female six-week-old BALB/C mice were immunised with three doses of 30 µg PfVFT1 protein at Day 0, 14, 21 subcutaneously. The first dose was formulated, at 1:1 volume ratio, with complete Freund’s adjuvant (Sigma-Aldrich), while the remaining two doses were formulated, at 1:1 volume ratio, with incomplete Freund’s adjuvant (Sigma-Aldrich). Three weeks following the last immunization, the mice were sacrificed, and the sera were harvested. Mice are anaesthetised using isoflurane (4% for induction, 1–2% for maintenance) and immunogen is administered through subcutaneous injection in the space between the skin and the underlying muscle. At the end of the experiment, the mice are euthanised using CO_2_ at a displacement rate of 30%. Experiments and procedures were approved by Institutional Animal Care and Use Committee (IACUC #140968), in accordance with the Agri-Food and Veterinary Authority (AVA) rules and National Advisory Committee for Laboratory Animal Research (NACLAR) of Singapore.

### Immunofluorescence

Parasite slides (liver sporozoite or blood stage parasites) were dried and fixed in cold methanol for 5 min. After blocking in 10% FBS for an hour, the slides were incubated with primary antibody (either mouse anti-PfVFT1, rabbit anti-CSP1, rabbit anti-Rh5^56^, rabbit anti-EBA175, or rabbit anti-MSP1 antibody) for an hour (diluted 1:200 in 10% FBS). Following a secondary antibody incubation for 30 min (Alexa Fluorophore-conjugated anti-mouse IgG or anti-rabbit IgG (Molecular Probes); diluted 1:1000), the slides were mounted using VectaShield Mounting Medium with DAPI (Vector Laboratories), covered with coverslips and viewed under fluorescence microscopy (Olympus FV1000 inverted confocal microscopy). Representative images from three independent experiments are shown.

### RNA expression

Blood stage parasites were harvested at various time points after invasion: (1) 2–6 h, (2) 10–16 h, (3) 18–22 h, (4) 26–30 h, (5) 34–38 h, (6) 42–46 h. DNA was digested extensively before RNA extraction using the RNeasy RNA isolation kit (Qiagen), according to manufacturer’s protocol. For cDNA synthesis, 1 µl of diluted RNA (10 ng/mL) and Primer PfVFT1F were used. The reverse transcription was performing using the SuperScript III reverse transcriptase (Thermo Fisher Scientific), according to manufacturer’s protocol. qRT-PCR was then performed in triplicates using TaqMan Fast Advanced Master mix (Thermo Fisher Scientific), according to manufacturer’s protocol.

Reaction mixture comprising of 1 µL of cDNA, 5 µL of TaqMan Fast Advanced Master mix, 1 µL of 9 µM forwards and reverse primer, 1 µL of 2.5 µM Taqman Probe (Supplementary Table 1, Thermo Fischer Scientific) and 1 µL of RNase-free water was prepared in 384-well plate and qRT-PCR was performed, using 7900HT Fast Real-Time PCR System (Applied Biosystems). Reaction conditions were set as the following: (a) 50 °C for 2 min; (b) 95 °C for 20 s; (c) 40 cycles of a 2-step reaction consisting of 95 °C for 2 s followed by 60 °C for 30 s. Upon completion of the run, data were analysed using the ΔΔCT method. Briefly, using the 2–6 h time point as the reference timepoint, ΔΔCT was calculated as ΔCt_timepoint_ – ΔCt_2-6 h_, with ΔCt determined as Ct_gene of interest_ – Ct_*ATL*_ (the latter, arginine-tRNA ligase, used as housekeeping gene). The fold change of PfVFT1 expression between reference time point (2–6 h) and the later time points was calculated as 2^−Δ ΔCT^. Expression was analysed in three independent experiments, with the mean fold change being plotted.

### Western blot

Recombinant PfVFT1 was loaded and run on pre-casted 4–12% Bis-Tris gels (Thermo Fisher Scientific) with MOPS buffer (Thermo Fisher Scientific). The antigen was then transferred onto nitrocellulose membrane using the iBlot 2 Dry Blotting system (Thermo Fisher Scientific). The membrane was then blocked with 5% low-fat milk (Sigma-Aldrich) for 1 h. Blocking was followed by primary antibody incubation (mouse anti-PfVFT1 sera or anti-hsp70, diluted 1:1000 in 10% FCS, 0.05% Tween20) for 1 h, and then secondary antibody incubation (HRP-conjugated goat anti-mouse IgG (Sigma-Aldrich), diluted 1:5000 in 10% FCS, 0.05% Tween20) for 30 min. The bands were visualised on HyperFilm ECL (GE Healthcare) using the Amersham ECL Prime western blotting detection reagent (GE Healthcare).

For parasite western blot to examine PfVFT1 protein expression, blood stage parasites (3D7) were harvested at ring (10–14 h), trophozoite (26–30 h) and schizonts (42–46 h) stages. Following three freeze/thaw cycles, the parasite pellet was lysed in lysis buffer (10 ml lysis buffer: 4 ml 10% SDS, 0.5 ml 10% Triton X-114, 5 ml 1x PBS, 0.5 ml water) for 15 min. Protease inhibitor (Roche) was added, and the parasite lysate was stored until use. Culture supernatant was harvested when the parasites were at schizonts (42–46 h) stage. 20 µl culture supernatant was loaded for western blot to examine if the PfVFT1 protein was secreted into the culture supernatant. For parasite western blot to verify mutants, blood stage parasites (3D7 and 3D7^∆PfVFT1^ mutants) were harvested at the schizont stage. Representative blots from three independent experiments were shown.

### ELISA

For antigen ELISA, recombinant PfVFT1 antigen was diluted in 100 mM bicarbonate/carbonate coating buffer to a final concentration of 5 µg/ml and coated onto 96-well plates overnight at 4 °C. Following an hour incubation with 10% FCS at 37 °C, the plates were incubated with serially diluted mouse sera (naïve or anti-PfVFT1) or diluted human sera (from both sera sets) for an hour at 37 °C. Secondary incubation (HRP-conjugated goat anti-mouse IgG or IgM and HRP-conjugated goat anti-human IgG or IgM, diluted 1:5000) was carried out for 30 min at 37 °C. All antibodies were diluted in 10% FCS. The plates were then developed with 1 mg/ml TMB substrate (SurModics). The reaction was stopped by 3 M sulfuric acid and then read at 450 nm (Perkin Elmer Enspire 2300). Mean values from three independent experiments were plotted.

For parasite ELISA, *P. falciparum* 3D7 iRBCs (at 5% parasitemia) were harvested. For one 96 well plate, 100 µl iRBCs (diluted to 1 ml with PBS) was frozen and thawed (a total of three freeze/thaw cycles) to allow RBC rupture. The parasites were collected by centrifugation after the thawing of the iRBCs. The parasite pellet was then resuspended in PBS and coated onto poly-L-lysine-coated 96 well plate. Following centrifugation of the plate, the parasite-coated plate was fixed and permeabilised with methanol for 10 min before blocking with 10% FCS for an hr at 37 °C. Primary and secondary antibody incubations, as well as substrate development, were similarly performed as in the antigen ELISA. Mean values from three independent experiments were plotted.

### *Plasmodium* parasite plasmid constructs

The ∆PfVFT1 mutant was generated using the CRISPR-Cas9 system^22^. The pUF1-Cas9 and pL6- *egfp* plasmids were kindly provided by Dr Jose-Juan Lopez-Rubio^22^. The pUF1-Cas9 plasmid encodes for the Cas9 protein that generates double-strands break. The pL6-*egfp* plasmid was used to clone homology regions.

To generate the pL6-*PfVFT1* plasmid, the *PfVFT1* homology regions, HR1 and HR2, were first amplified using genomic DNA as template and the primer pairs B3nHR1_F and B3nHR1_R, and B3nHR2_F and B3nHR2_R, respectively (Table S1). The homology regions were then cloned into the pL6 plasmids using the restriction enzyme sites AflII/SpeI and NcoI/EcoRI. Following the cloning of the homology regions, the pL6-*PfVFT1* plasmid was used to clone the *PfVFT1* guide DNA to generate the pL7-*PfVFT1* plasmid. The guide RNA in the pL7-*PfVFT1* plasmid guide the Cas9 to cause double-strands break in targeted sites and donor DNA, which is drug resistant cassette flanked by *PfVFT1* homology regions, to facilitate homologous recombination. The pL7-*PfVFT1* plasmid was generated by first gel-extracting the BtgZI-digested pL6-*PfVFT1* plasmid and then replacing the BtgZI-adaptor with the guide DNA sequence using in-Fusion cloning kit. Using the mutant genomic DNA as template and primer pairs (P1 and P4, P2 and P3, P1 and P2; Table S1), PCR analysis showed *PfVFT1* gene disruption and integration of the resistance cassette through a double-crossover recombination (Fig. S5).

Gene complementation of *PfVFT1* in the ∆PfVFT1 mutant was performed via episomal insertion. The *PfVFT1* gene was amplified and cloned into pBCam-3HA using the restriction enzyme sites NcoI/BamHI to generate the recombinant pBCam-PfVFT1-3HA plasmid. Using the extracted DNA from the complemented mutant as template and primer pairs pCamF and pCamR, PCR analysis confirmed the presence of the *PfVFT1* gene in the complemented mutant (Fig. S8).

All PCR amplifications were performed with Phusion High Fidelity DNA polymerase (Thermo Fisher Scientific), according to manufacturer’s protocol, with the exception of the elongation temperature of 68 °C. Ligation was performed using either T4 DNA ligase (Fermentas) or In-Fusion cloning kit, according to manufacturer’s protocol. Genomic DNA extraction from iRBCs, PCR purification, reaction cleanup and gel extraction were performed using DNeasy blood and tissue kit (Qiagen), QIAquick PCR purification kit (Qiagen), MinElute Reaction Cleanup kit (Qiagen), and QIAquick gel extraction kit (Qiagen), respectively, according to manufacturer’s protocol.

### *Plasmodium* parasite transfection

Synchronous asexual blood-stage 3D7 cultures or 3D7^∆PfVFT1^ mutant, obtaining by sorbitol treatment and MACS-sorting, were transfected by electroporating ring-stage parasites using 4D Nucleofector (Lonza) and the Amaxa P3 Primary cell 4D Nucleofector kit (Lonza). Briefly, for each reaction, 100 µl of iRBCs, at 10% parasitemia, were washed with incomplete cytomix (120 mM KCl, 0.15 mM CaCl_2_, 2 mM EGTA, 5 mM MgCl_2_, 10 mM K_2_HPO_4_/KH_2_PO_4_, 25 mM HEPES, pH 7.6), and resuspended in DNA mix, before transferring into nucleocuvettes for electroporation. The iRBCs were then washed with pre-warmed culture media, supplemented with 400 µl fresh RBC and fresh pre-warmed culture media (final haematocrit 5%). Following 24 h incubation at 37 °C, 5% CO_2_, culture supernatant was replaced with fresh culture media supplemented with 50 nM pyrimethamine for ∆PfVFT1 mutant generation, or with 0.48 µM blasticidin for complementation of the ∆PfVFT1 mutant. Selection was done by changing the media every 2 days. Analysis of the mutants was performed 14–21 days post transfection/selection.

### Reinvasion inhibition assay

*P. falciparum* 3D7 reinvasion inhibition assays (schizonts) were performed as previously described^57^. Normal human red blood cells (RBCs) were stained with CFSE (12 μM; Sigma) and mixed with MACS (Miltenyi)-sorted schizonts in 96 well plates (Becton Dickinson) at a final parasitemia of 1% and final haematocrit of 2%. The heat-inactivated were then added at a final dilution of 1:20. After 24 h of incubation at 37 °C, 5% CO_2_, the cultures were stained with 8 µM of Hoechst 33342 (Sigma). Parasitemia of each well was determined by flow cytometry. Newly reinvaded RBCs were defined as CFSE and Hoechst double positive RBCs. RBC reinvasion inhibition efficiency was defined as the ratio of the subtraction of parasitemia in the test well (test antibody, anti-PfVFT1 or anti-ompF (ompF is a membrane protein on *Escherichia coli*)) from the parasitemia in the control well (naïve serum) to the parasitemia in the control well, expressed as a percentage. Three independent experiments were performed, with the mean percentage being plotted.

Merozoite reinvasion inhibition assays were performed similarly, except that merozoites were used in place of schizonts. The preparation of merozoites from schizonts was as previously described^58^. MACS-sorted schizonts were filtered through a 1.2 µm Acrodisc syringe filter. The concentration of the merozoites and CFSE-stained RBCs was determined using CountBright Absolute Counting beads (Thermo Fisher Scientific), according to manufacturer’s protocol. The merozoite reinvasion assay was set up with a merozoites:RBCs ratio of 5:1 and a final haematocrit of 2%, in triplicate. Three independent experiments were performed, with the mean percentage being plotted. For analysis of reinvasion assays, unpaired t-tests were used to compare groups, unless stated otherwise.

### Schizont arrest assay

*P. falciparum* 3D7 schizont arrest assays were performed as previously described^17^. RBCs were mixed with MACS-sorted early schizonts in 96 well microtiter plates at a final parasitemia of 3% and final haematocrit of 2%. The heat-inactivated sera were added at a final dilution of 1:20. The plate was then incubated at 37 °C, 5% CO_2_. Smears were performed at the start of the assay, 0 h, and at 12 h. The percentage of schizonts arrested at 12 h was calculated by the number of schizonts present at 12 h divided by the number of schizonts present at 0 h, multiplied by 100. For each well, 200 parasite-infected RBCs were counted. Three independent experiments were performed, with the mean percentage being plotted. Mann-Whitney U tests were used to compare groups.

### Parasite growth curve

To study the replication curves of the 3D7^∆PfVFT1^ mutants, freshly thawed parasite cultures were set up with a final parasitemia of 3% and a final haematocrit of 3%. Every two days (at ring stage), the parasite culture media were replaced with fresh media and the parasite cultures were diluted to a final parasitemia of 3%. Smears were performed daily and parasitemia was measured. For each smear, 1000 parasites were counted, using the Olympus digital microscopy DP21. Mean parasitaemia from three independent experiments was plotted.

To examine the growth of the 3D7^∆PfVFT1^ mutants through the different stages of blood cycle, freshly thawed parasite cultures (a final parasitemia of either 3% or 0.2%, and a final haematocrit of 3%) were followed for 104 h. Smears were prepared every 4 h. The relative abundance of the ring, trophozoite and schizont stages was calculated. For each smear, 1000 parasites were counted, using the Olympus digital microscopy DP21. Independently repeated cell analysis cycle was plotted in Supplementary Fig. 7.

### Merozoite count

Freshly thawed parasite cultures (a final parasitemia of 3% and a final haematocrit of 3%) were followed till the schizont stage (42–46 h), before the culture media were replaced by fresh media supplemented with 10 µM E64 (Sigma) for 12 h. Following the 12 h incubation, smears were prepared, and the number of merzoite per schizont was enumerated. A total of 200 schizonts were counted for each parasite culture. Mean values from three independent experiments were plotted. Mann-Whitney U tests were used to compare groups.

### Parasite phagocytosis

Parasite phagocytosis was performed using either the schizont^59^ or the merozoite^60^ stage. Adherent RAW264.7 mouse macrophage was seeded in 48-well plate at 1 × 10^5^ cells/well 12 h before the phagocytosis assay.

For schizont phagocytosis assays^59^, MACS-sorted schizonts were stained with 12 μM CFSE, washed to remove any residual CFSE and then enumerated using CountBright Absolute Counting beads. The enumerated CFSE-stained iRBCs (schizonts) were first preincubated with sera (at final dilutions of 1:10, 1:50, 1:200 and 1:1000) for 30 min at room temperature before the addition of RAW264.7 cells. Sera are either naïve mouse sera or mouse anti-PfVFT1 sera. RAW264.7 was then added to the mixture (consisting of CFSE-stained parasites and the antibody mix) at a iRBC:RAW264.7 ratio of 25:1. After an hour incubation at 37 °C, 5% CO_2_, the culture supernatant was removed and the RAW264.7 cells were harvested. Non-phagocytosed parasites were lysed in ammonium chloride lysing solution (15 mM NH_4_Cl, 10 mM NaHCO_3_, 1 mM EDTA) for 3 min. Cells were then washed twice with cold 5% FCS before FACS analysis on LSRII flow cytometer (Becton Dickinson). Viable cells were gated on forward and side scatter. Phagocytosis was defined as the percentage of CFSE-positive cells (RAW264.7 cells with internalised CFSE-stained parasites). Mean values from three independent experiments were plotted. Mann-Whitney U tests were used to compare groups.

For merozoite phagocytosis assays^60^, MACS-sorted schizonts were filtered through a 1.2 µm Acrodisc syringe filter to obtain the merozoites. The merozoites were stained with CFSE and enumerated using CountBright Absolute Counting beads, and then preincubated in sera (at final dilutions of 1:10, 1:50, 1:200 and 1:1000). Sera are either naïve mouse sera or mouse anti-PfVFT1 sera. RAW264.7 was then added to the mixture (consisting of CFSE-stained parasites and the antibody mix) at a merozoite:RAW264.7 ratio of 10:1. Phagocytosis was defined as the percentage of CFSE-positive cells (RAW264.7 cells with internalised CFSE-stained parasites). Mean values from three independent experiments were plotted. Mann-Whitney U tests were used to compare groups.

### Antibody-dependent cellular inhibition

ADCI assays were performed as previously described^61^. RAW264.7 mouse macrophage was seeded in 24-well plate at 2 × 10^5^ cells/well 12 h before the assay. On the day of the assay, MACS-sorted schizonts were added to the wells at 0.5% parasitemia and 2% haematocrit, and sera were added at final dilutions of 1:10, 1:50, 1:200 and 1:1000. Sera are either mouse anti-MSP1 or mouse anti-PfVFT1 sera. Fresh RAW264.7 culture media were added in 50 µl aliquots at 48 and 72 h. At 86 h, the trophozoites in the culture media were harvested, stained with 8 µM Hoechst and FITC-conjugated anti-mouse CD45 (BD Biosciences) and analysed on LSRII flow cytometry. Parasitemia was defined as Hoechst-positive and FITC-negative events. The specific growth inhibitory index (SGI) was calculated as the percentage inhibition relative to that for the control sera. Mean values from three independent experiments were plotted. Paired t-tests were used to compare groups.

### Complement deposition

Complement deposition ELISA assays were performed as previously described^54^. For the C1q deposition assay, 96-well plates were coated with merozoites, obtained by filtering MACS-sorted schizonts through a 1.2 µm Acrodisc syringe filter, at 5 × 10^6^ merozoite/well overnight. Following the overnight incubation, the merozoite-coated plates were washed with PBS. the merozoite-coated plates were blocked with 10% FCS and then incubated first with sera (either naïve mouse or mouse anti-PfVFT1, diluted 1:100). After washing off the unbound sera, recombinant C1q (10 µg/ml, Merck) were then added. After washing off the unbound C1q, C1q was detected using HRP-conjugated anti-C1q antibodies (diluted 1:1000, Thermo Fisher Scientific). For the C5-C9 deposition assay, the merozoites were first incubated with the sera (diluted 1:100, naïve or anti-PfVFT1) and either 25% normal human serum (NHS) or heat-inactivated normal human serum (HI NHS) for 10 min at 37 °C, before being coated onto the 96-well plates. Following the overnight incubation, the coated plates were washed with PBS. The plates were then blocked with 10% FC. C5-C9 was detected using rabbit anti-C5-C9 (diluted 1:1000, Abcam) and HRP-conjugated goat anti-rabbit antibodies (diluted 1:1000, Sigma-Aldrich). Mean values from three independent experiments were plotted. Unpaired t-test was used to compare groups.

### Merozoite lysis

Merozoite lysis assays were performed as previously described^54^. Merozoites were added into 96well plates at 5 × 10^6^ merozoite/well, and incubated with sera (either naïve mouse or mouse anti-PfVFT1, diluted 1:20) and either 25% NHS or HI NHS for 10 min at 37 °C. Samples were taken at 0, 1, 2, 3, 4, 5 and 10 min, and immediately diluted 1:100 with cold 1% FCS. Merozoites in the samples were counted using CountBright Absolute Counting beads. Mean values from three independent experiments were plotted. Kruskal–Wallis test was used to compare groups.

### Antibody-dependent respiratory burst

Antibody-dependent respiratory burst assays were performed as previously described^33^, with modifications. The assays utilized mouse neutrophils and merozoites.

To prepare the effector cells, female 6 weeks old C57BL/6 mice were sacrificed, and the femur and tibia were removed. Bone marrow cells were flushed out of the bone shafts, and the bone marrow-derived neutrophils were isolated from the bone marrow cells using the mouse neutrophil isolation kit (Miltenyi Biotec), according to manufacturer’s protocol. Viability of the isolated neutrophils was confirmed by trypan blue exclusion, and purity was confirmed by CD11b-positive and Ly-6G-postive staining, using e660-conjugated anti-CD11b (eBiosciences) and PE-conjugated anti-Ly-6G antibodies (BioLegend) and LSRII flow cytometry.

For the parasite preparation, MACS-sorted schizonts were filtered through a 1.2 µm Acrodisc syringe filter to obtain the merozoites. Merozoites were counted using CountBright Absolute Counting beads and frozen till use.

On the day of the assay, 96-well black with clear bottomed plates (Corning) were coated with poly-L-lysine (Sigma-Aldrich) and washed with water before the addition of the thawed merozoites (following three freeze/thaw cycles) to the well at 2 × 10^6^ merozoite/well. The plate was blocked with casein block solution (Thermo Fisher Scientific) for an hour and incubated with diluted sera (1:20, 1:50, 1:200, 1:1000, naïve mouse or mouse anti-PfVFT1 sera) for an hour. Within 2 min of the final PBS wash, 50 µl isoluminol (0.04 mg/ml, Sigma-Aldrich) and 50 µl isolated neutrophils (1 × 10^7^/ml) were added to each well. Luminescence (in relative light unit, rlu) was read immediately every min for an hour using Perkin Elmer Enspire 2300. 100 µM phorbol myristate acetate (PMA, Sigma–Aldrich) was used as positive control. The experiment was performed in triplicates. Mean values from three independent experiments were plotted. Mann-Whitney U tests were used to compare groups.

### PfVFT1 genetic variation analysis

PfVFT1 sequence alignment results were obtained directly from www.plasmodb.com^39^. In addition, DNA was isolated from 90 field isolates (Maesot, Thailand). The *PfVFT1* gene was PCR-amplified with the primer pair PfVFT1sqPCR_F and PfVFT1sqPCR_R. The PCR products were then purified and sequenced using primer PfVFT1sqF. The DNA sequences were translated to protein sequences using https://web.expasy.org/translate/. The protein sequences were then aligned using https://www.ebi.ac.uk/Tools/msa/clustalo/.

### Statistical analysis

Statistical analyses were performed using Graph Pad Prism (5.1). *p* < 0.05 is considered significant.

## Supplementary information

Supplementary Information

## Data Availability

The data generated in this study can be obtained upon reasonable request to the corresponding author. DNA sequences from 90 Thai field isolates are deposited in Genbank, and the Genbank accession numbers are PX636225 to PX636314, respectively.
