# Supplementary material for: Vaccine-induced mouse antibodies targeting Plasmodium falciparum PfVFT antigen inhibit blood stages through multiple mechanisms
Source: NPJ Vaccines. 2026 Mar 30;11:107. doi: 10.1038/s41541-026-01433-9 (PMC13199436; doi:10.1038/s41541-026-01433-9)
Supplement: Supplementary file 1 — Supplementary Information [file 41541_2026_1433_MOESM1_ESM.pdf]

Supplementary Table 1

For	Primer name	Sequence (5' to 3')
RT-PCR	PfVFT1F	GTGGAAGAAGTAGTAAATAATAAGAGCA
qPCR (PfVFT)	PfVFT1probe	TCGATCAATCTGGAAATAATCTACCT
	PfVFT1qF	TTTATCGAATTCACGTCTTG
	PfVFT1qR	TTGGTAACCCACTTGTTAGTTT
qPCR (PfATL)	PfATLprobe	TGCATGTTGGTCATTTACGCTCAACT
	PfATLqF	ATGGTAATGTACTGGTTGATTT
	PfATLqR	ACATGATTAACTCGATGGGT
rPfVFT1 cloning	rPfVFT1cdF	AAAAAGCTTATGATGCACATCTTCTGCAAA
	rPfVFT1cdR	AAAGGATCCTCACTTGAAGTAGGCGCTATG
Parasite constructs - HR1	B3nHR1_F	TTTCCGCGGGGAGGACTAGTAGACCAAATAATAACTATAAAAAGGG
	B3nHR1_R	TTACAAAATGCTTAAGTATTAATCGCTTGCTCTTATTATTTAC
Parasite constructs - HR2	B3nHR2_F	ATTTATTAAATCTAGAATTCAACCTAGAGGATCTTAAATCATATATTTC
	B3nHR2_R	TTTTACCGTTCCATGGAAGTATGCAGAGTGTCCCGT
Guide DNA	Guide DNA_F	CATATTAAGTATATAATATTGTCAATTCTTTAACGGCAGCAGTTTTAGAGCTAGAA
	Guide DNA_R	TTCTAGCTCTAAAACTGCTGCCGTTAAAGAATTGACAATATTATATACTTAATATG
Parasite transfection verification	P1	AGACCAAATAATAACTATAAAAAGGG
	P2	TGCCATTTCATATTAAATAGTATATCA
	P3	GGGATAGCGATTTTTTTACTGTC
	P4	TTTTGTTTCTATAAATTGATATCTTAATT
Parasite mutant sequencing	PfVFT1sqPCR_F	AGACCAAATAATAACTATAAAAAGGG
	PfVFT1sqPCR_R	TGGAACCAAATAATAATCAATTTG
	PfVFT1sqF	CCACGCTTACATTGATATACACAT
Parasite mutant complementation verification	pCamF	GGATCCATGATGCATATTTTTTGC
	pCamR	CTGGAACATCGTAAGGATACG
	cPfVFT1sq_R1	CATAACATCAGGCCAAAAACC

Complemented parasite mutant sequencing	cPfVFT1sq_R2	CTGGAACATCGTAAGGATACG

Supplementary Table 2

Antigen library #	plasmodb	Rationale for selection	References
PfL140	PF3D7_1134300	Associated with protection in Tanzanian children	Raj DK et al., 2014[1]
PfL141	PF3D7_1136200	Associated with protection in Kenyan children	Osier FH et al., 2014[2]
PfL142 (PfVFT1)	PF3D7_0606800		
PfL143	PF3D7_0830400	Surface localisation	Khosh-Naucke M et al., 2018[3]
PfL144	PF3D7_1010100	Associated with protection in in humans by immunization with radiation-attenuated sporozoites	Aguiar et al., 2015[4]
PfL145	PF3D7_0313200		
PfL146	PF3D7_0323600		
PfL147	PF3D7_0511400		
PfL148	PF3D7_1014100		
PfL149	PF3D7_0404800		

Supplementary Figures

a

Antigen library #	plasmodb	Nucleotide position on coding sequence (CDS)	Transfection efficiency	Infectious bites									Non-infectious bites				
				1	2	3	4	5	6	7	8	9	10	11	12	13	14
PfL140	PF3D7_1134300	3546-5412	87%														
PfL141	PF3D7_1136200	58-1968	37%														
PfL142 (PfVFT1)	PF3D7_0606800	67-897	30%														
PfL143	PF3D7_0830400	58-414	44%														
PfL144	PF3D7_1010100	1-867	11%														
PfL145	PF3D7_0313200	1-675	12%														
PfL146	PF3D7_0323600	1-1626	74%														
PfL147	PF3D7_0511400	1-1014	27%														
PfL148	PF3D7_1014100	1045-2874	11%														
PfL149	PF3D7_0404800	1-1530	79%														

b

Antigen library #	plasmodb	Nucleotide position on coding sequence (CDS)	Transfection efficiency	Infectious bites									Non-infectious bites				
				1	2	3	4	5	6	7	8	9	10	11	12	13	14
PfL140	PF3D7_1134300	3546-5412	87%														
PfL141	PF3D7_1136200	58-1968	37%														
PfL142 (PfVFT1)	PF3D7_0606800	67-897	30%														
PfL143	PF3D7_0830400	58-414	44%														
PfL144	PF3D7_1010100	1-867	11%														
PfL145	PF3D7_0313200	1-675	12%														
PfL146	PF3D7_0323600	1-1626	74%														
PfL147	PF3D7_0511400	1-1014	27%														
PfL148	PF3D7_1014100	1045-2874	11%														
PfL149	PF3D7_0404800	1-1530	79%														

c

Antigen library #	plasmodb	Nucleotide position on coding sequence (CDS)	Transfection efficiency	Infectious bites					Non-infectious bites			
				1	2	3	4	5	6	7	8	9
PfL140	PF3D7_1134300	3546-5412	87%									
PfL141	PF3D7_1136200	58-1968	37%									
PfL142 (PfVFT1)	PF3D7_0606800	67-897	30%									
PfL143	PF3D7_0830400	58-414	44%									
PfL144	PF3D7_1010100	1-867	11%									
PfL145	PF3D7_0313200	1-675	12%									
PfL146	PF3D7_0323600	1-1626	74%									
PfL147	PF3D7_0511400	1-1014	27%									
PfL148	PF3D7_1014100	1045-2874	11%									
PfL149	PF3D7_0404800	1-1530	79%									

d

Antigen library #	plasmodb	Nucleotide position on coding sequence (CDS)	Transfection efficiency	Infectious bites					Non-infectious bites			
				1	2	3	4	5	6	7	8	9
PfL140	PF3D7_1134300	3546-5412	87%									
PfL141	PF3D7_1136200	58-1968	37%									
PfL142 (PfVFT1)	PF3D7_0606800	67-897	30%									
PfL143	PF3D7_0830400	58-414	44%									
PfL144	PF3D7_0220000	1-867	11%									
PfL145	PF3D7_0220000	1-675	12%									
PfL146	PF3D7_1147800	1-1626	74%									
PfL147	PF3D7_1147800	1-1014	27%									
PfL148	PF3D7_1334800	1045-2874	11%									
PfL149	PF3D7_0508000	1-1530	79%									

Supplementary Fig. 1. Antibody response against 10 hypothetical antigens. IgG (a, c) and IgM (b, d) profile for individuals that were exposed to either infectious or non-infectious mosquito bites (chloroquine sera set 1 (a, b) and 2 (c, d)). All 10 constructs corresponding to 10 antigen genes are shown here. Each of the individual serum was incubated with transfected cells that expressed the indicated individual *P. falciparum* antigen on their surface. The population of cells that binds sera antibodies was determined via flow cytometry. Positive serum response (where the serum response is above 5%) was indicated by coloured boxes (green or blue).

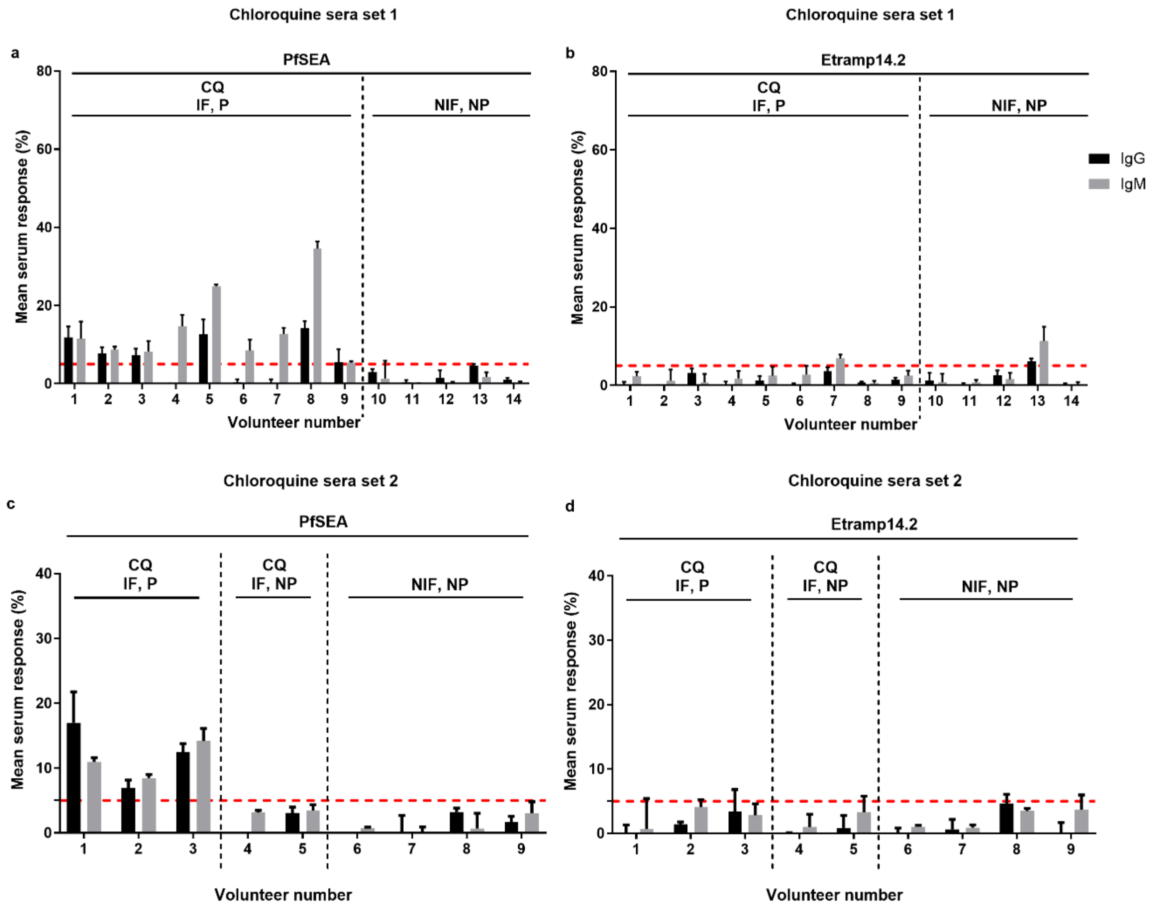

Supplementary Fig. 2. Antibody response against PfSEA1 and Etramp14.2. IgG (a, c) and IgM (b, d) profile for individuals that were exposed to either infectious or non-infectious mosquito bites (chloroquine sera set 1 (a, b) and 2 (c, d)). Each of the individual serum was incubated with transfected cells that expressed the indicated individual *P. falciparum* antigen on their surface. The population of cells that binds sera antibodies was determined via flow cytometry. Serum response above 5% was defined as positive serum response, indicated by the red dotted horizontal line. Serum was analysed in three independent experiments (three technical experiment repeats), with the mean antibody response being plotted. Error bar represents standard deviation. CQ: chloroquine; IF: infective bites; NIF: non-infective bites; P: protected; NP: non-protected.

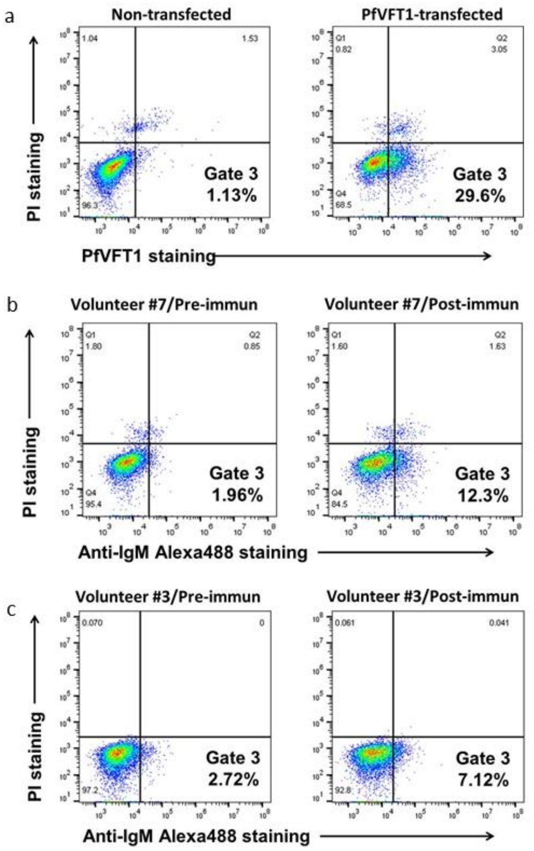

Supplementary Fig. 3. Transfection efficiency and antibody response. (a) Transfection efficiency was defined as Alexa Fluor 488-positive (PfVFT1 staining) and PI-negative labelling, Q3. Gates on non-transfected cells were applied to transfected cells. The transfection efficiency of the Pf antigen-transfected cells shown here, PfVFT1, was 29.6%. (b) and (c) Antibody response, which was defined by Alexa Fluor 488-positive (IgM shown here) and PI-negative labelling (Q3), was gated on negative controls (pre-immunised sera, pre-immun), and applied to the post-immunised sera (post-immun). Antibody response for volunteer #7 in the chloroquine sera set 1 was shown in (b). As an example, the volunteer's reactivity against PfVFT1 post immunisation was 12.3%, as indicated in Q3. The pre-immunisation baseline for the volunteer is 1.96%, as indicated in Q3. The PfVFT1 reactivity for this volunteer was calculated as 12.3-1.96=10.34% then normalised to the transfection efficiency, whereby (10.34/29.6)*100 gave an

antigen antibody response of 34.9%. The antibody response for volunteer #3 in the chloroquine sera set 2 was shown in (c).

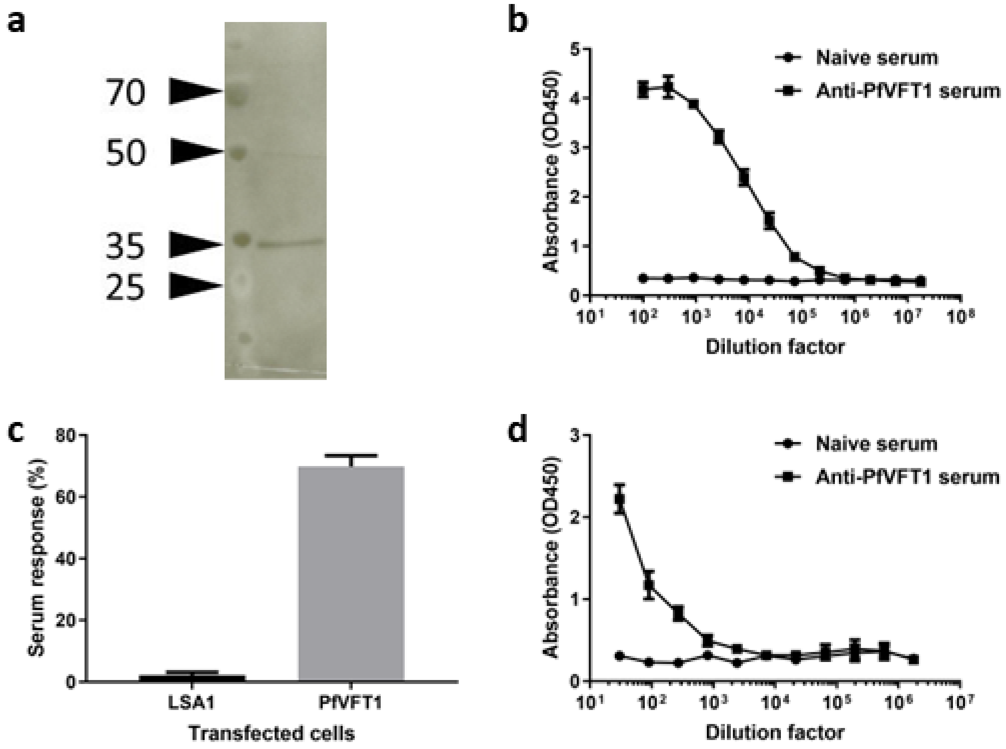

Supplementary Fig. 4. PfVFT1 specificity of mouse anti-PfVFT1 serum. (a) Purified PfVFT1 antigen was loaded onto protein gel and probed with the mouse anti-PfVFT1 serum. One single band of ~35 kDa, corresponding to the size of the PfVFT1 antigen, was observed. (b) PfVFT1 antigen ELISA was performed with mouse pooled naïve and pooled anti-PfVFT1 sera**.** Antigen binding was observed with the mouse anti-PfVFT1 serum. Serum IgG was analysed in three independent experiments (three technical experiment repeats), with the mean antibody response being plotted. Error bar represents standard deviation. (c) PfVFT1-transfected cells, were probed with mouse pooled naïve and pooled anti-PfVFT1 sera. Positive IgG binding was observed with the mouse anti-PfVFT1 serum. Serum was analysed in three independent experiments (three technical experiment repeats), with the mean antibody response being plotted. Error bar represents standard deviation. (d) Blood stage parasites (schizont stage) were coated onto

96wells plates and probed with mouse pooled naïve and pooled anti-PfVFT1 sera**.** Antigen binding was observed with the mouse pooled anti-PfVFT1 serum. Serum IgG was analysed in three independent experiments (three technical experiment repeats), with the mean antibody response being plotted. Error bar represents standard deviation.

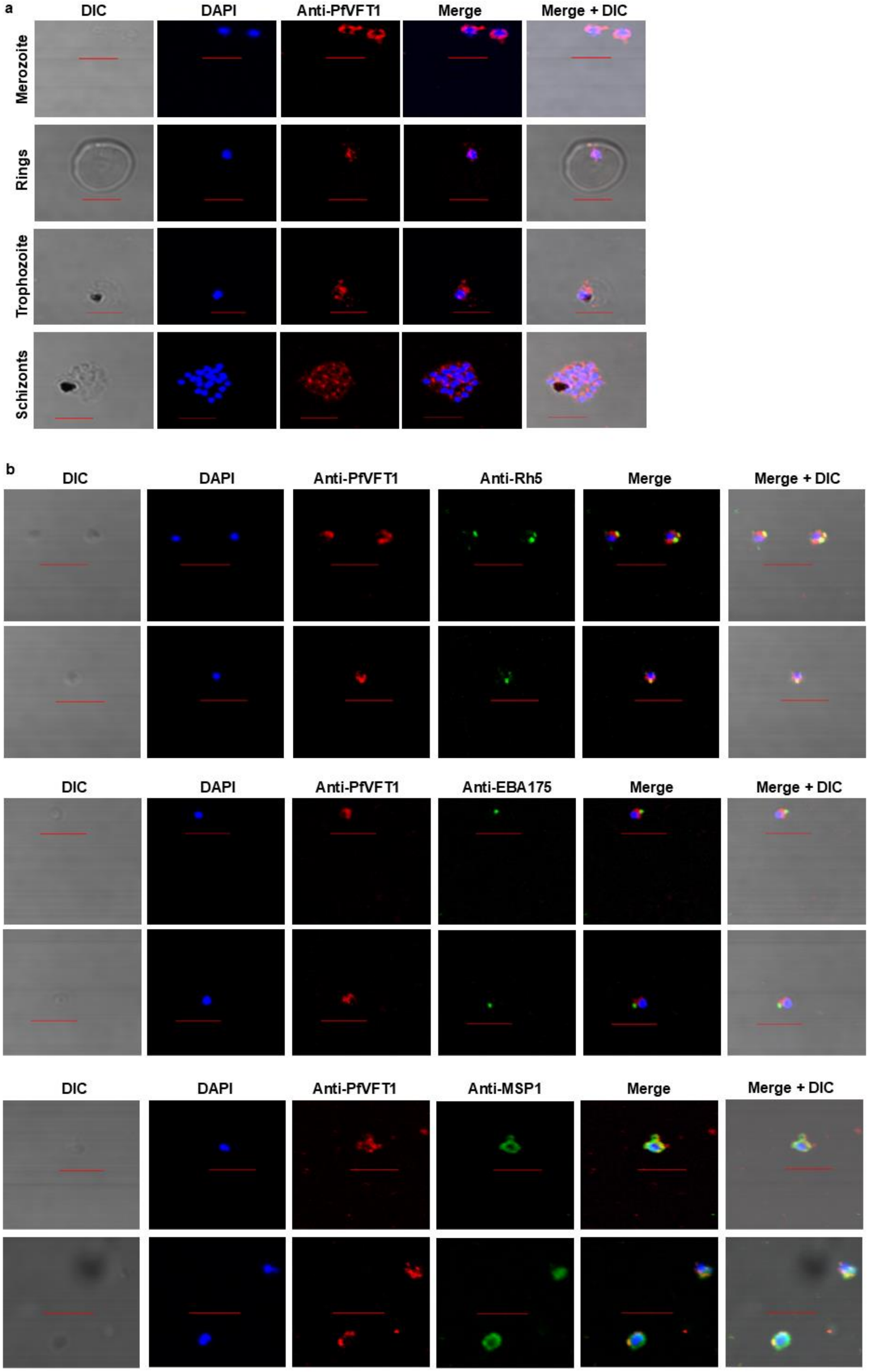
a
DIC
DAPI
Anti-PfVFT1
Merge
Merge + DIC
Merozoite
Rings
Trophozoite
Schizonts
b
DIC
DAPI
Anti-PfVFT1
Anti-Rh5
Merge
Merge + DIC
DIC
DAPI
Anti-PfVFT1
Anti-EBA175
Merge
Merge + DIC
DIC
DAPI
Anti-PfVFT1
Anti-MSP1
Merge
Merge + DIC

Supplementary Fig. 5. PfVFT1 antigen staining on parasites. (a) Blood stages parasite slides were prepared for the merozoite, ring, trophozoite and schizont stage. The slides were fixed and stained with pooled anti-PfVFT1 sera. Red bar represents 5 µm**.** (b) Merozoite slides were fixed and stained with pooled anti-PfVFT1 sera (indicated by the green signal), in addition to one of the following sera, Rh5, EBA175 or MSP1 (indicated by the red signal). Red bar represents 5 µm**.**

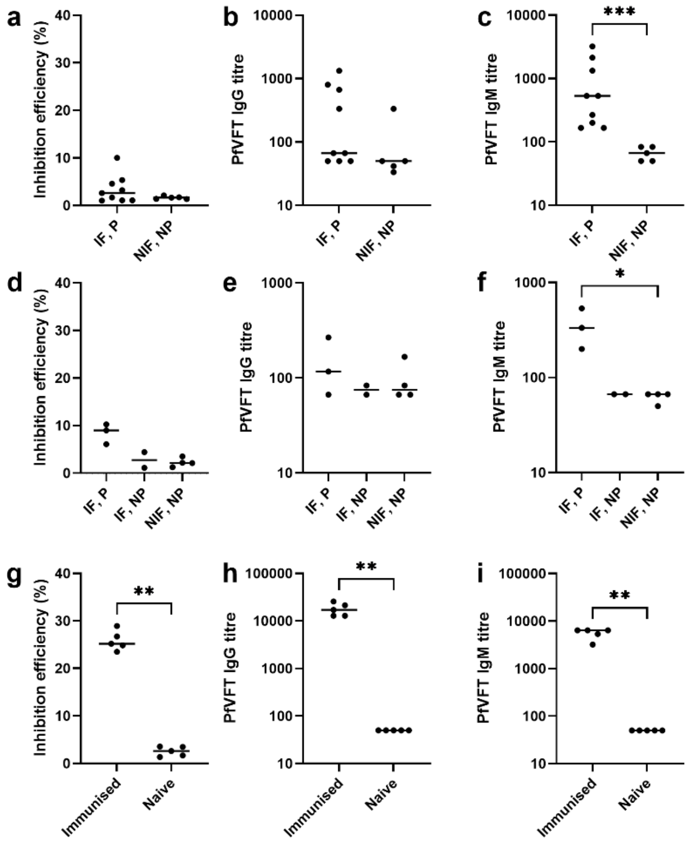

Supplementary Fig. 6. Reinvasion inhibition capability of sera from human vaccinees. *P. falciparum* schizont reinvasion assays using (a) chloroquine sera set 1, (d) chloroquine sera set 2, (g) mouse pooled naïve or pooled anti-PfVFT1 sera. Each of the three independent experiments (three technical experiment repeats), with a merozoites : RBCs ratio of 5:1, a final haematocrit of 2%. and 1:20 serum dilution. Inhibition efficiency was defined as the ratio of the subtraction of parasitemia in the test well from the parasitemia in the control well to the parasitemia in the control well, expressed as a percentage. Values above 0% indicated positive inhibition (no invasion) while values below 0% indicated no inhibition (positive invasion). Mean reinvasion inhibition efficiency values from three independent experiments were plotted. Each dot represents each individual/mouse, with error bars indicating standard deviation. Mann-Whitney

U tests were used to compare groups. PfVFT1 antigen IgG and IgM ELISA was performed with sera from human vaccinees in (b, c) chloroquine set 1 and (e, f) chloroquine set 2, and (h, i) mouse pooled naïve or pooled anti-PfVFT1 sera**.** Serum was analysed in three independent experiments (three technical experiment repeats), with the mean antibody titre for each serum being plotted. Titre refers to the lowest dilution that gave a positive signal above the secondary-antibody-control (no serum control). Error bar represents standard deviation.

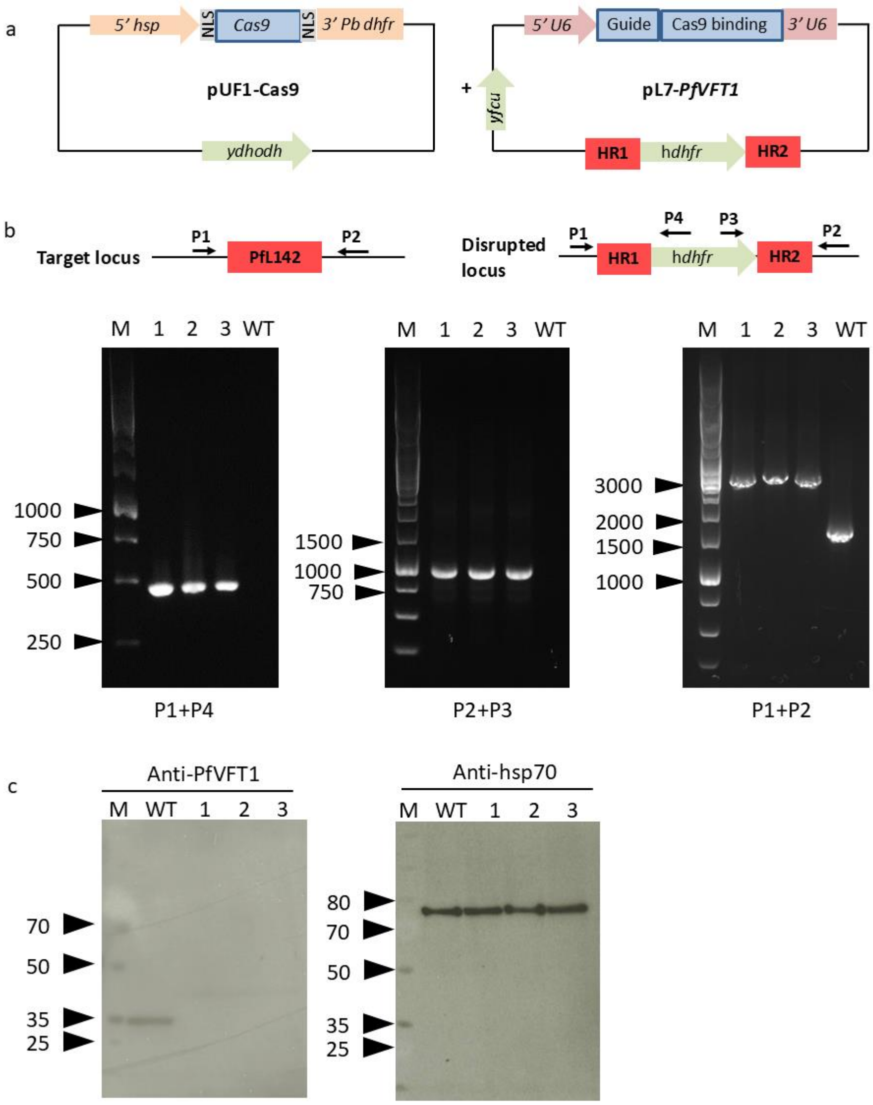

Supplementary Fig. 7. Generation of $3D7^{\Delta PfVFT1}$ mutants. $3D7^{\Delta PfVFT1}$ mutants were generated using the CRISPR/Cas9 system. Blood stage 3D7 parasites were transfected with the pUF1-Cas9 plasmid and the pL7-*PfVFT1* plasmid (a). Analysis of the mutants was performed 14-21 days post transfection/selection. Using genomic DNA of the $3D7^{\Delta PfVFT1}$ mutants as template, PCR analysis (b) showed *PfVFT1* gene disruption and integration of the resistance cassette through a double-crossover recombination. M: DNA ladder; 1, 2, 3: three clones of $3D7^{\Delta PfVFT1}$ mutants, namely 1E9, 3H12, 5E10; WT: the parental isolate, 3D7. (c) Blood stage parasites were

harvested at schizont stage, lysed to obtain parasite lysate and analysed by western blot. The absence of PfVFT1 protein expression by the 3D7$^{\Delta PfVFT1}$ mutants was confirmed. Anti-hsp70 staining served as positive control for loading.

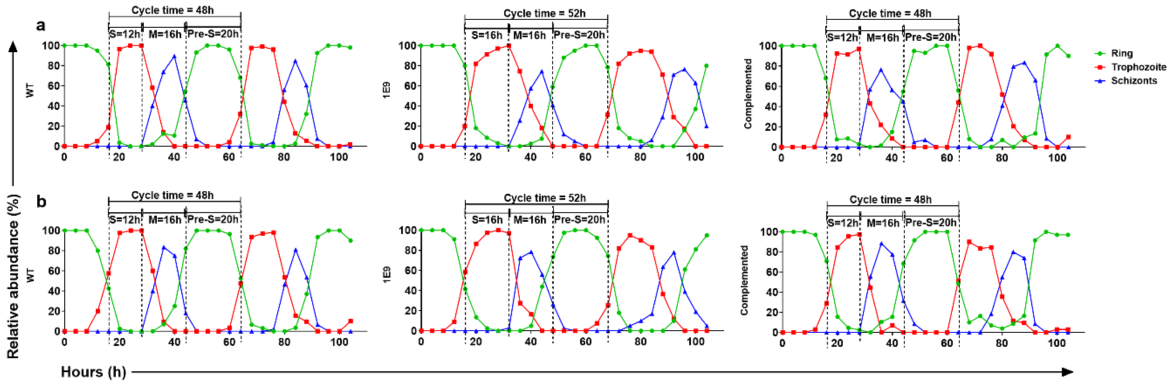

Supplementary Fig. 8. Cell cycle analysis of the 3D7$^{\Delta PfVFT1}$ 1E9 mutant and complemented 3D7$^{\Delta PfVFT1}$ 1E9 mutant. Parasite cultures of either (a) a final parasitemia of 3% or (b) a final parasitemia of 0.2%, and a final haematocrit of 3%, were followed for 104 hr. Smears were prepared every 4 hr. For each smear, 1000 parasites were counted. The relative abundance of the ring, trophozoite and schizont stages was calculated.

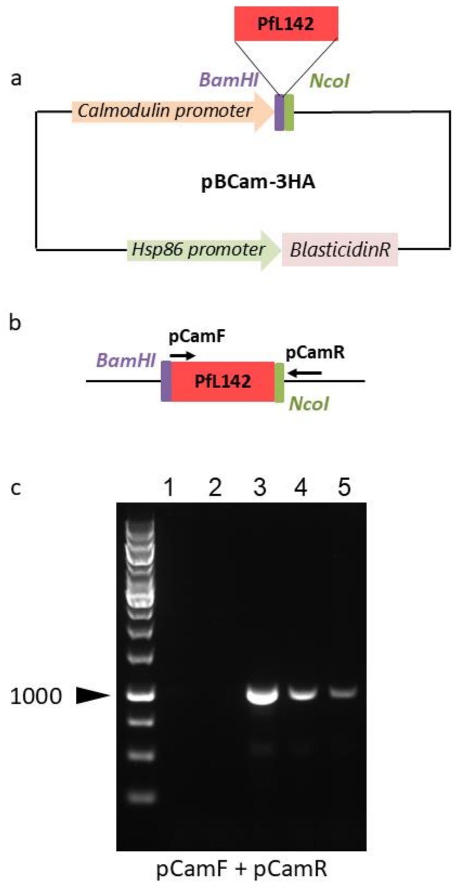

Supplementary Fig. 9. Gene complementation of $3D7^{\Delta PfVFT1}$ mutants. Gene complementation was performed through (a) episomal insertion via pBCam-3HA plasmid. Blood stage $3D7^{\Delta PfVFT1}$ 1E9 mutant parasites were transfected with the recombinant pBCam-PfVFT1-3HA plasmid. (b) Verification of recombinant pBCam-PfVFT1-3HA plasmid was performed using primer pair pCamF and pCamR. Analysis of the mutants was performed 14-21 days post transfection/selection. (c) PCR analysis of the complemented $3D7^{\Delta PfVFT1}$ 1E9 mutant was performed using the extracted DNA from the complemented mutant as template and primer pairs pCamF and pCamR. M: DNA ladder; 1: $3D7^{\Delta PfVFT1}$ 1E9; 2: empty pBCam-3HA plasmid, 3: recombinant pBCam-PfVFT1-3HA plasmid; 4: pBCam-PfVFT1-3HA-transfected $3D7^{\Delta PfVFT1}$ 1E9; 5: 3D7.

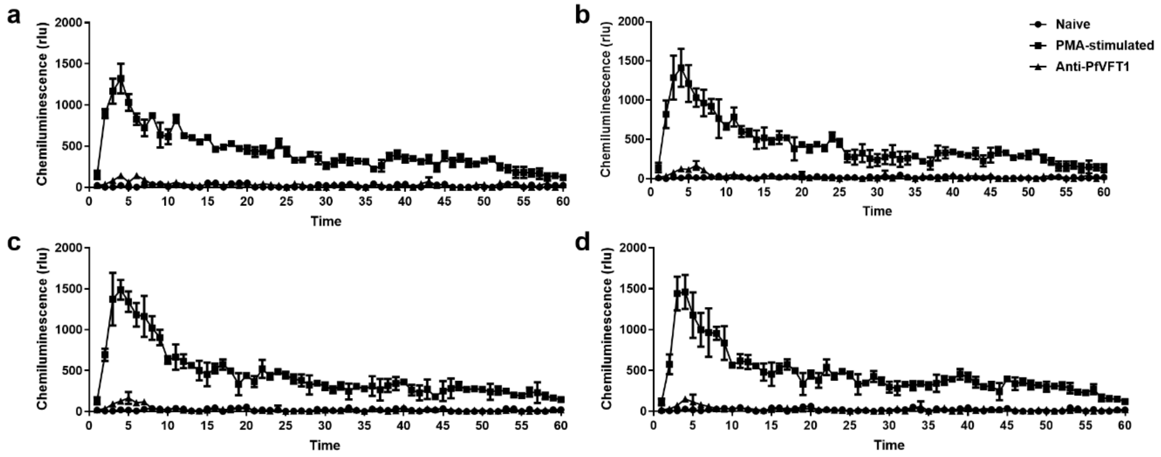

Supplementary Fig. 10. Antibody-dependent respiratory burst. Thawed merozoites (following three freeze/thaw cycles) were coated onto the 96well black with clear bottomed plates at 2x10^6 merozoite/well. Following blocking with casein block solution, it was incubated with diluted sera (pooled naïve mouse or pooled mouse anti-PfVFT1 sera) at the following dilution: (a) 1:20, (b) 1:50, (c) 1:200, (d) 1:1000. Isoluminol (0.04 mg/ml, Sigma-Aldrich) and isolated mouse neutrophils (1x10^7/ml) were then added to each well, and the luminescence (in relative light unit, rlu) was read immediately every min for an hour. 100 µM phorbol myristate acetate (PMA) was used as positive control. Mean values from three independent experiments (three technical experiment repeats) were plotted. Mann-Whitney U tests were used to compare groups.

a

Source	Isolates aligned	Non-coding SNPs	Synonymous SNPs	NonSynonymous SNPs
Plasmodb.com	202	0	7	4

b

1 - MMHIFCKLFLFFSFVYISNI - 20 281 - HKINVFDSFLKTGHSAYFK - 299

F F L I

d

Source	Isolates sequenced	Non-coding SNPs	Synonymous SNPs	NonSynonymous SNPs
Maesot, Thailand	90	0	1	0

Supplementary Fig. 11. PfVFT1 sequence analysis. (a) Genetic variation of PfVFT1 for 202 isolates, as reported by plasmodb.com. (b) Sequence segment, indicating the four non-synonymous SNPs reported by plasmodb.com. Number represents the amino acid position. The SNPs are highlighted in red. (c) Genetic variation of PfVFT1 for 90 isolates from Maesot, Thailand.

Supplementary Fig. 12 – uncropped version of blot in Fig. 2d

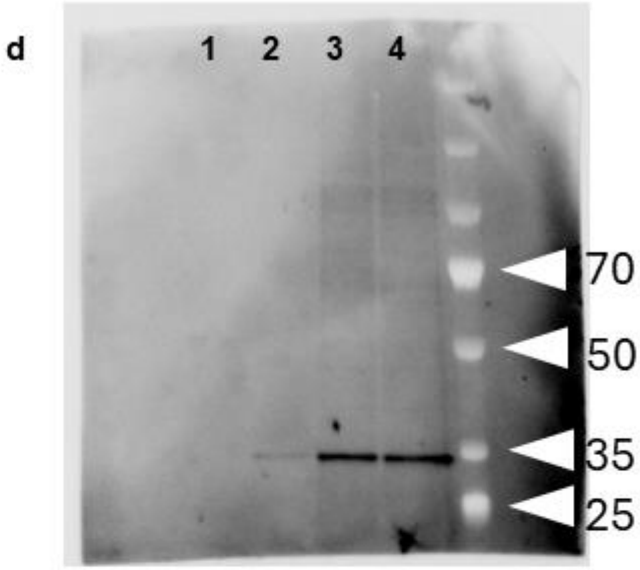

138 **Supplementary Fig. 13 – uncropped version of blot in Supplementary Fig. 4a**

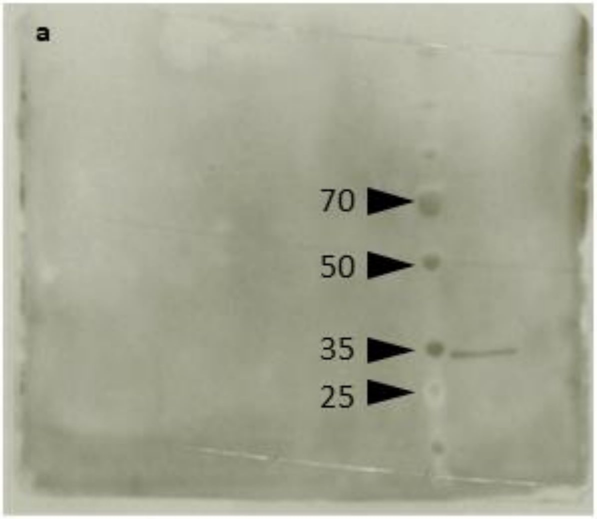

139

140

Supplementary Fig. 14 – uncropped version of blot in Supplementary Fig. 7c

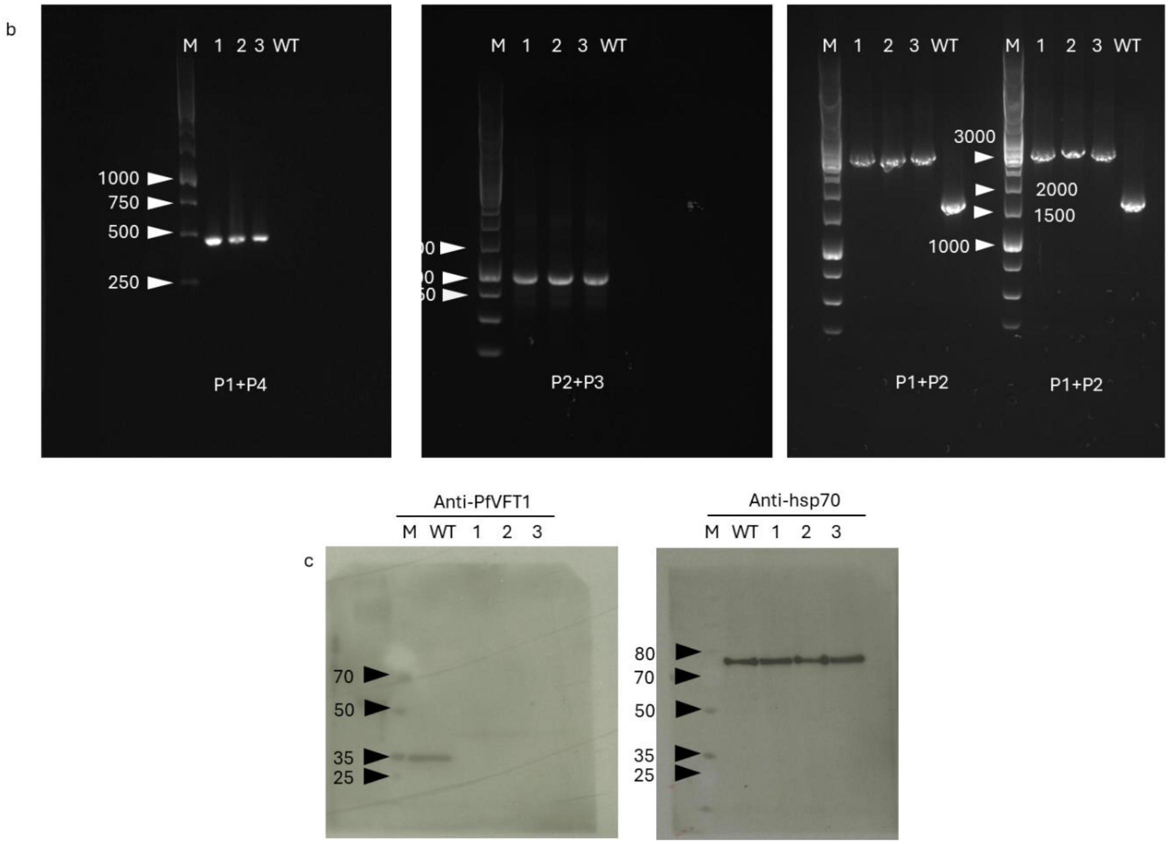

Supplementary Fig. 15 – uncropped version of blot in Supplementary Fig. 9c

c

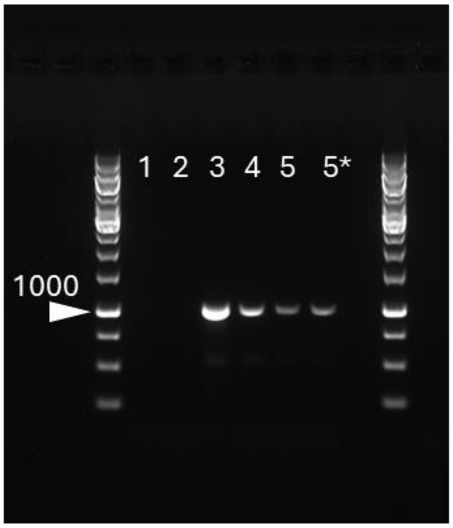

* Duplicate of lane 5

Supplementary References

1. Raj DK, *et al.* Antibodies to PfSEA-1 block parasite egress from RBCs and protect against malaria infection. *Science* **344**, 871-877 (2014).

2. Osier FH, *et al.* New antigens for a multicomponent blood-stage malaria vaccine. *Sci Transl Med* **6**, 247ra102 (2014).

3. Khosh-Naucke M, *et al.* Identification of novel parasitophorous vacuole proteins in P. falciparum parasites using BioID. *Int J Med Microbiol* **308**, 13-24 (2018).

4. Aguiar JC, *et al.* Discovery of Novel Plasmodium falciparum Pre-Erythrocytic Antigens for Vaccine Development. *PLoS One* **10**, e0136109 (2015).
